# Differentiation of Pathogenic Th17 Cells Is Negatively Regulated by Let-7 MicroRNAs in a Mouse Model of Multiple Sclerosis

**DOI:** 10.3389/fimmu.2019.03125

**Published:** 2020-01-17

**Authors:** Constance C. Angelou, Alexandria C. Wells, Jyothi Vijayaraghavan, Carey E. Dougan, Rebecca Lawlor, Elizabeth Iverson, Vanja Lazarevic, Motoko Y. Kimura, Shelly R. Peyton, Lisa M. Minter, Barbara A. Osborne, Elena L. Pobezinskaya, Leonid A. Pobezinsky

**Affiliations:** ^1^Department of Veterinary and Animal Sciences, University of Massachusetts, Amherst, MA, United States; ^2^Department of Chemical Engineering, University of Massachusetts, Amherst, MA, United States; ^3^Experimental Immunology Branch, National Cancer Institute, National Institutes of Health, Bethesda, MD, United States; ^4^Department of Immunology, Graduate School of Medicine, Chiba University, Chiba, Japan

**Keywords:** EAE, CD4, miRNA, IL-1R1, IL-23R, CCR2, CCR5

## Abstract

Multiple sclerosis (MS) is a disabling demyelinating autoimmune disorder of the central nervous system (CNS) which is driven by IL-23- and IL-1β-induced autoreactive Th17 cells that traffic to the CNS and secrete proinflammatory cytokines. Th17 pathogenicity in MS has been correlated with the dysregulation of microRNA (miRNA) expression, and specific miRNAs have been shown to promote the pathogenic Th17 phenotype. In the present study, we demonstrate, using the animal model of MS, experimental autoimmune encephalomyelitis (EAE), that let-7 miRNAs confer protection against EAE by negatively regulating the proliferation, differentiation and chemokine-mediated migration of pathogenic Th17 cells to the CNS. Specifically, we found that let-7 miRNAs may directly target the cytokine receptors *Il1r1* and *Il23r*, as well as the chemokine receptors *Ccr2* and *Ccr5*. Therefore, our results identify a novel regulatory role for let-7 miRNAs in pathogenic Th17 differentiation during EAE development, suggesting a promising therapeutic application for disease treatment.

## Introduction

Multiple sclerosis (MS) is a chronic inflammatory disease of the central nervous system (CNS) that affects ~2.5 million people worldwide, with a strong predominance in women (1). The animal model of neuroinflammation, experimental autoimmune encephalomyelitis (EAE), recapitulates the pathological and clinical symptoms of MS and has been extensively used to study this disorder (2). In both MS and EAE, autoreactive CD4^+^ type-17 helper T (Th17) cells that are generated by exposure to IL-23 and IL-1β (3–5) migrate to the CNS and cross the blood-brain barrier by following gradients of chemokines secreted by CNS-resident innate lymphoid cells (6). The transcription factor Bhlhe40, which is induced in encephalitogenic Th17 by IL-1β signaling, positively regulates the secretion of granulocyte-macrophage colony-stimulating factor (GM-CSF) (7–9). GM-CSF is a proinflammatory cytokine essential for disease induction as it promotes the activation, differentiation, and recruitment of monocytes and dendritic cells to the CNS, as well as the mobilization of the local microglia (5, 10–14). In turn, GM-CSF-stimulated glial and dendritic cells secrete IL-6 and IL-23, thereby reinforcing the differentiation and maintenance of pathogenic Th17 cells (15). Inflammatory myeloid cells produce reactive oxygen species and cytokines that cause neuron demyelination and axonal damage, which leads to the disruption of neuronal signaling, eventually resulting in disabling physical symptoms, including progressive loss of motor function, which reflect the extent of neurodegeneration (1).

About a third of the total risk factors for MS development can be attributed to genetic variation, and genome-wide association studies have identified more than 100 different genetic variants associated with MS and related autoimmune disorders (16–19). Many of these susceptibility factors consist of variants of genes which are involved in Th17 pathways and contain single nucleotide polymorphisms within the untranslated regions (UTRs) of their messenger RNA (mRNA). Given that UTR sequences are targeted by factors controlling mRNA translation and stability (20), the post-transcriptional dysregulation of these genes may be responsible for the aberrant cytokine responsiveness and effector function observed in autoreactive Th17 cells.

MicroRNA (miRNA)-mediated RNA interference is one of the most well-studied post-transcriptional mechanisms that potently regulates gene expression. MiRNAs are short non-coding RNAs that bind target mRNAs in a sequence-specific manner to inhibit translation by inducing target mRNA degradation or ribosome stalling (21). It has been estimated that miRNAs control the expression of approximately one third of the total human gene pool, including genes involved in immune cell differentiation. In fact, altered miRNA expression has been shown in encephalitogenic Th17 cells from active MS lesions, and specific upregulated miRNAs, such as miR-155 and miR-326, were demonstrated to promote the pathogenic Th17 phenotype (22–25).

The lethal-7 (let-7) family of miRNAs comprises multiple members and is highly represented in lymphocytes (26). As such, many lymphocyte subsets have been shown to be dependent on let-7 miRNAs for their development, homeostasis, and differentiation. For instance, the upregulation of let-7 miRNAs during natural killer T (NKT) cell development in the thymus is necessary for the differentiation of IFNγ-producing effectors (27). In the periphery, let-7 miRNA expression is required for the survival and maintenance of the quiescent phenotype in naïve T cells (28, 29). During lymphocyte activation, let-7 miRNAs have been shown to inhibit the metabolic reprogramming of both B cells and CD8^+^ T cells by negatively regulating the expression of the transcription factor c-Myc and the enzyme hexokinase 2 (28, 30). Moreover, let-7 miRNAs have been shown to play a regulatory role in CD8^+^ cytotoxic T lymphocyte (CTL) function by directly targeting the transcription factor Eomesodermin, as well as in CD4^+^ helper T cell responses during HIV infection and airway inflammation by regulating the expression of the cytokines IL-10 and IL-13, respectively (28, 31–33). One study also proposed a regulatory role for let-7f expression in Th17 cell function in women with severe asthma, which was controlled by ovarian sex hormone levels (34). In addition, the exosome-mediated transfer of let-7 miRNAs to various immune cells has been proposed as a suppressive mechanism used by regulatory T (Treg) cells, and has also been reported to inhibit the generation and function of Treg cells (35, 36). Yet, the precise contribution of let-7 miRNAs in MS pathogenesis and autoreactive Th17 cell differentiation has been controversial (25). Disease-promoting roles have been proposed for specific let-7 miRNA family members (23, 36, 37), but other reports have suggested that let-7 miRNA expression may confer protection against MS (38, 39).

Here, we investigate the role of let-7 miRNAs in the generation of pathogenic Th17 cells. We demonstrate that the differentiation of Th17 cells in EAE requires the downregulation of let-7 miRNAs in naïve CD4^+^ T cells upon antigen stimulation. Specifically, we found that high let-7 miRNA expression in activated CD4^+^ T cells prevents EAE development by inhibiting the clonal expansion, IL23R/IL-1R1-dependent acquisition of pathogenic function, and CCR2/CCR5-dependent chemokine-mediated migration of Th17 cells to the CNS, while depletion of let-7 miRNAs enhances Th17 cell pathogenicity and aggravates EAE. Therefore, let-7 miRNA delivery to pathogenic Th17 cells may be a promising therapeutic strategy for the treatment of autoimmune diseases such as MS.

## Results

### Let-7 Expression in CD4^+^ T Cells Inhibits EAE Development

The role of let-7 miRNAs in the differentiation of pathogenic CD4^+^ T cells remains unclear (23, 25, 36–39). Previously, we have shown that let-7 miRNA expression in CD8^+^ T cells prevents the differentiation of CTLs and must be downregulated during antigen stimulation (28). Here, we found that, similarly to CD8^+^ T cells, naïve CD4^+^ T cells expressed high levels of let-7, which were rapidly downregulated upon activation, proportionally to both the duration (Figure S1A) and strength of TCR stimulation (Figure S1B). Based on these observations, we hypothesized that let-7 miRNAs inhibit the differentiation of CD4^+^ T cells and may therefore compromise the generation of pathogenic Th17 cells and suppress the development of autoimmune disorders.

**Figure 1:**
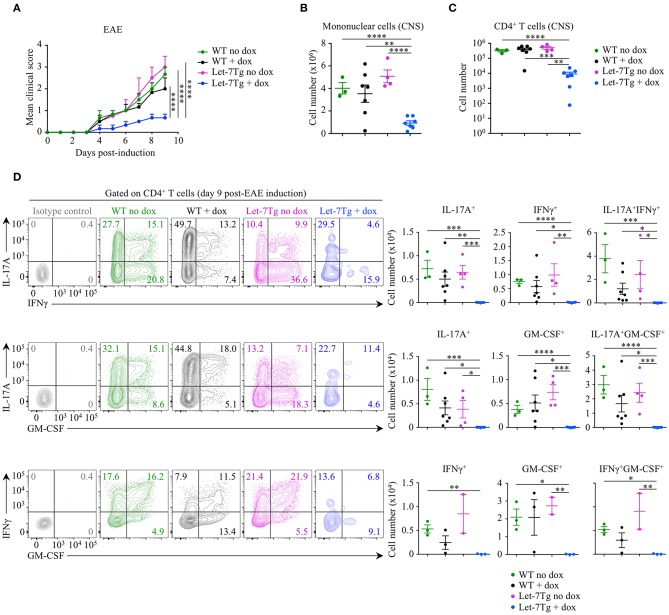
Downregulation of let-7 miRNAs upon activation is required for CD4^+^ T cell pathogenicity in EAE. **(A)** Mean clinical scores in vehicle- (no dox) treated wild-type (WT) (*n* = 3) and Let-7Tg (*n* = 4) mice or doxycycline- (+ dox) treated WT (*n* = 7) and Let-7Tg (*n* = 7) mice immunized with MOG_35−55_ in complete Freund's adjuvant (CFA) and pertussis toxin (60 ng). **(B)** Number of total mononuclear cells at the peak of the disease (day 9–15 post-immunization) in the CNS of vehicle- (no dox) or doxycycline- (+ dox) treated WT vs. Let-7Tg mice. **(C)** Number of CNS-infiltrated CD4^+^ T cells at the peak of the disease (day 9–15 post-immunization) in vehicle- (no dox) or doxycycline- (+ dox) treated WT vs. Let-7Tg mice as analyzed by flow cytometry. **(D)** Intracellular staining of CD4^+^ T cells from the CNS of vehicle- (no dox) or doxycycline- (+ dox) treated WT vs. Let-7Tg mice (left). Numbers indicate the frequencies of cytokine-positive cells within the indicated gates. Quantification of the numbers of cytokine-positive cells as assessed by flow cytometry for each staining strategy (right). *p < 0.05, **p < 0.01; ***p < 0.001, ****p < 0.0001 **(A–D)**, employing two-way ANOVA (A) or compared with WT using two-tailed Student's *t*-test **(B–D)**. Data are from two combined independent experiments (**A–C**; mean ± S.E.M. of each population from all mice), or from one experiment representative of two independent experiments (**D**; mean ± S.E.M. of each population from all mice).

To test our hypothesis *in vivo*, we acquired mice with a doxycycline-inducible let-7g transgene (Let-7Tg) in order to maintain high let-7g expression in activated CD4^+^ T cells (40). We used EAE susceptibility as a readout of CD4^+^ T cell differentiation in doxycycline- or vehicle-treated Let-7Tg and WT control mice immunized with the peptide antigen myelin oligodendrocyte glycoprotein, residues 35–55 (MOG_35−55_), in complete Freund's adjuvant (CFA). Only doxycycline-treated Let-7Tg mice developed a significantly milder disease in comparison to control mice (Figure 1A). Strikingly, the number of mononuclear cells (Figure 1B) and CD4^+^ T lymphocytes (Figure 1C) that infiltrated the CNS was strongly diminished in these mice. Overall, the frequencies and numbers of cytokine-producing CD4^+^ T cells were greatly reduced in the CNS of doxycycline-treated Let-7Tg mice (Figure 1D). In addition, *in vitro* MOG_35−55_-restimulated splenocytes from the same mice secreted less IL-17, IFNγ, and GM-CSF in comparison to that of control mice (Figure S1C). We obtained similar results using WT and Let-7Tg mice on a 2D2 RAG2-deficient (2D2Rag2KO) background, in which all CD4^+^ T cells express the 2D2 transgenic T cell receptor that recognizes the MOG_35−55_ peptide (41) (Figure S2). To assess whether the absence of let-7 miRNAs in CD4^+^ T cells leads to aggravated EAE, we used Lin28 transgenic mice (Lin28Tg) with T-cell specific ectopic overexpression of the fetal protein LIN28B that blocks let-7 miRNA biogenesis (27, 42–44). 2D2Rag2KO Lin28Tg mice developed stronger symptoms of EAE, where the phenotype of cytokine-producing pathogenic CD4^+^ T cells was enhanced even though T cell infiltration into the CNS was unchanged in comparison to controls (Figure S2), suggesting that let-7 miRNAs inhibit EAE development.

**Figure 2:**
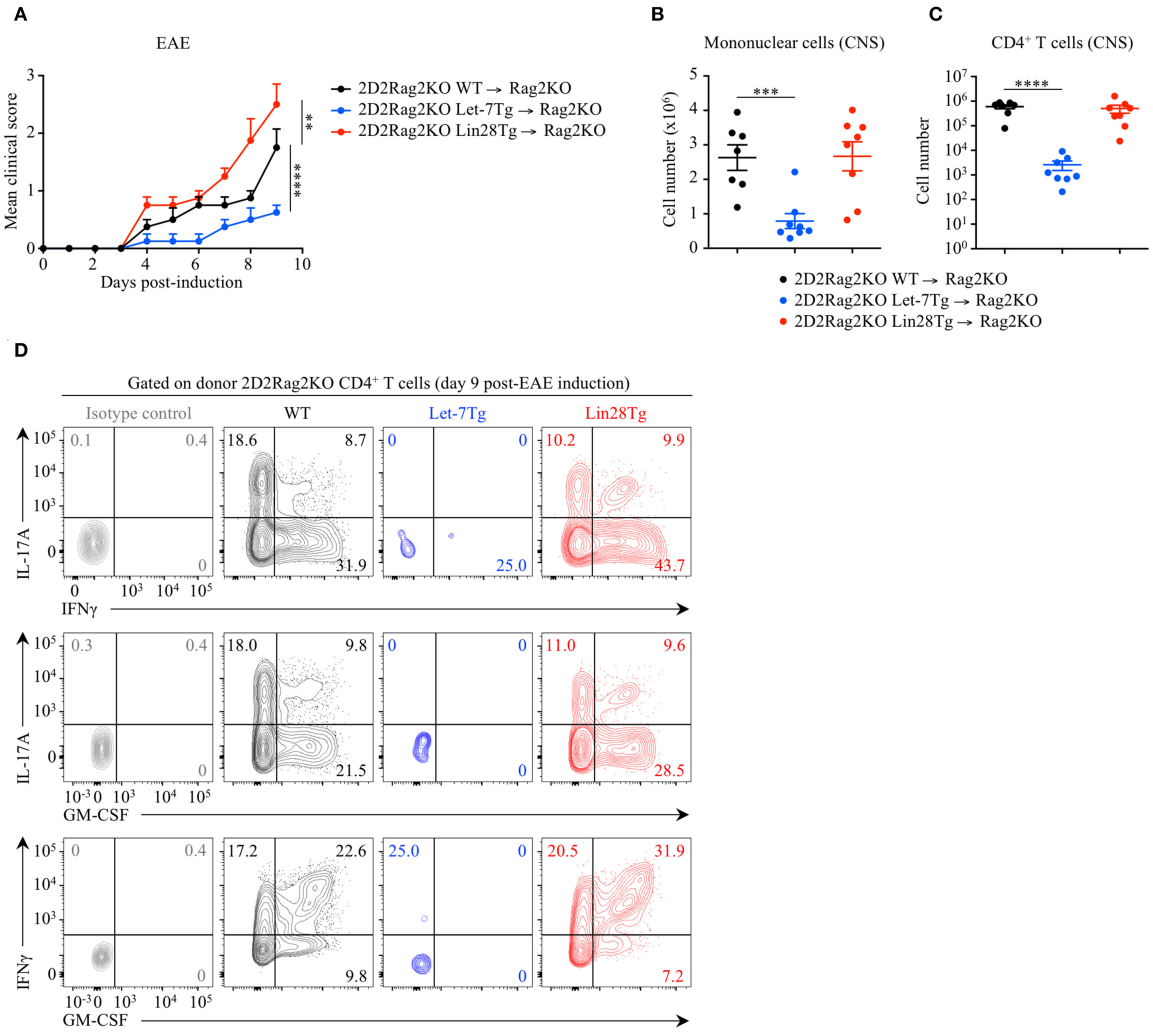
Let-7 miRNAs negatively regulate CD4^+^ T cell pathogenicity in a cell-intrinsic manner in EAE. **(A)** Mean clinical scores in Rag2KO recipient mice that received 2D2Rag2KO WT (*n* = 7), 2D2Rag2KO Let-7Tg (*n* = 7) or 2D2Rag2KO Lin28Tg (*n* = 8) naïve CD4^+^ T cells (2–2.5 × 10^6^ cells/recipient) and that were subsequently immunized with MOG_35−55_ in complete Freund's adjuvant (CFA) and pertussis toxin (60 ng). **(B)** Number of total mononuclear cells at the peak of the disease (day 9 post-immunization) in the CNS of Rag2KO recipients that received 2D2Rag2KO WT, 2D2Rag2KO Let-7Tg, and 2D2Rag2KO Lin28Tg cells. **(C)** Number of CNS-infiltrated 2D2Rag2KO CD4+ T cells at the peak of the disease (day 9 post-immunization) in Rag2KO recipients transferred with 2D2Rag2KO WT, 2D2Rag2KO Let-7Tg, and 2D2Rag2KO Lin28Tg cells as analyzed by flow cytometry. **(D)** Intracellular staining of donor CD4^+^ T cells from the CNS of Rag2KO recipients that received 2D2Rag2KO WT, 2D2Rag2KO Let-7Tg, and 2D2Rag2KO Lin28Tg cells (left). Numbers indicate the frequencies of cytokine-positive cells within the indicated gates. **p < 0.01, ***p < 0.001, ****p < 0.0001 **(A–C)**, compared with WT employing two-way ANOVA **(A)** or using two-tailed Student's *t*-test **(B,C)**. Data are from two combined independent experiments (**A–C**; mean ± S.E.M. of each population from all mice) or from one experiment representative of two independent experiments **(D)**.

To determine whether the let-7 miRNA-mediated protection against EAE is CD4^+^ T cell-intrinsic, we adoptively transferred naïve 2D2Rag2KO CD4^+^ T cells from Let-7Tg, Lin28Tg and control mice, into Rag2KO recipient mice that were subsequently immunized with MOG_35−55_ in CFA. At day 9 post-EAE induction, disease outcome (Figure 2A), CNS infiltration (Figure 2B), as well as frequencies and numbers of cytokine-producing donor 2D2Rag2KO CD4^+^ T cells that had differentiated into pathogenic CD4^+^ T cells (Figures 2C,D) recapitulated the results from our previous EAE experiments (Figure 1 and Figure S2). These results demonstrate that let-7 abolishes the development of EAE in a CD4^+^ T cell-intrinsic manner.

Although Let-7Tg CD4^+^ T cells were largely absent in the CNS of EAE-induced mice, they were found in the spleen, albeit at lower numbers than control cells (Figure S3). To explain this phenotype, we proposed four potential mechanisms: (1) poor cell survival, (2) reduced proliferation, (3) compromised differentiation, or (4) impaired trafficking.

### Let-7 Promotes Survival but Restricts the Proliferation of Activated CD4^+^ T Lymphocytes

**Figure 3:**
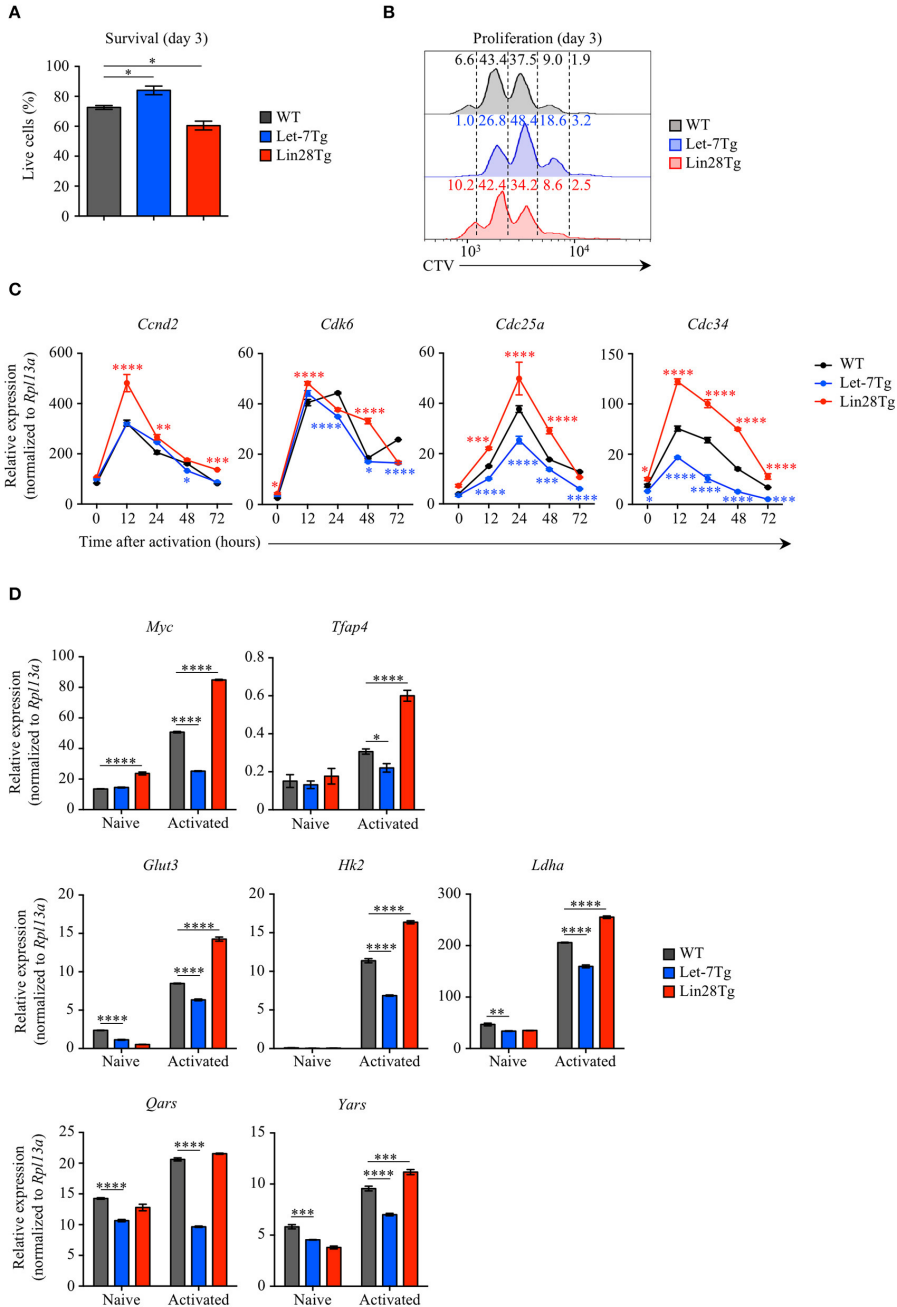
Let-7 miRNAs control the proliferation of CD4^+^ T cells by negatively regulating metabolic reprogramming and cell cycle progression. **(A)** Survival rate of WT, Let-7Tg and Lin28Tg CD4^+^ T cells activated *in vitro* for 3 days with α-CD3 and α-CD28 mAbs (5 μg/mL each) as analyzed by trypan blue exclusion. **(B)** Proliferation of Cell-Trace Violet-labeled WT, Let-7Tg and Lin28Tg CD4^+^ T cells activated *in vitro* for 3 days α-CD3 and α-CD28 mAbs (5 μg/mL each) as analyzed by flow cytometry. Numbers indicate the cell frequencies within the indicated gates for each genotype. **(C)** Quantitative RT-PCR analysis of the cell cycle regulators, cyclin D2 (*Ccnd2*), cyclin-dependent kinase 6 (*Cdk6*), cell division cycle 25a phosphatase (*Cdc25a*), and ubiquitin-conjugating enzyme E2 Cdc34 (*Cdc34*) in naïve CD4^+^ T cells activated with plate-bound α-CD3 and α-CD28 mAbs (5 μg/mL each) for increasing time periods as indicated, presented relative to results obtained for the ribosomal protein Rpl13a (control). **(D)** Quantitative RT-PCR analysis of the transcription factors Myc (*Myc*) and AP-4 (*Tfap4*), as well as Myc direct target genes involved in glycolysis and protein synthesis, glucose transporter 3 (*Glut3*), hexokinase 2 (*Hk2*), lactate dehydrogenase A (*Ldha*), glutamyl-tRNA synthetase (*Qars*) and tyrosyl-tRNA synthetase (*Yars*) in naïve CD4^+^ T cells activated with plate-bound α-CD3 mAbs and α-CD28 mAbs (5 μg/mL each) for 48 h, presented relative to results obtained for the ribosomal protein Rpl13a (control). *p < 0.05, **p < 0.01; ***p < 0.001, ****p < 0.0001 **(A,C,D)**, compared with WT using two-tailed Student's *t*-test. Data are from one experiment representative of at least two independent experiments (**A,C,D**; mean ± S.E.M. of technical triplicates of each population from all mice) or from two independent experiments **(B)**.

To examine whether let-7 miRNAs negatively regulate the survival of activated CD4^+^ T cells during EAE, we measured the survival rate of Let-7Tg, Lin28Tg, and WT control CD4^+^ T cells activated *in vitro* for 3 days. Interestingly, Let-7Tg cells exhibited better survival than their WT counterparts, while the recovery of Lin28Tg lymphocytes was significantly lower (Figure 3A). In fact, these results are in agreement with our recently published findings in naïve CD4 and CD8 T cells (29), suggesting that let-7 expression also supports the survival of activated CD4^+^ T cells. Thus, the reduced numbers of CNS-infiltrated Let-7Tg CD4^+^ T cells recovered during EAE cannot be explained by increased cell death.

Based on our data in CD8^+^ T cells (28), we tested whether let-7 miRNAs can suppress CD4^+^ T cell proliferation. To address this question, Let-7Tg, Lin28Tg, and WT control naïve CD4^+^ T cells were labeled with CellTrace Violet (CTV) and activated *in vitro*. We observed that Let-7Tg CD4^+^ T cells proliferated less than activated control T cells, while Lin28Tg CD4^+^ T lymphocytes proliferated more (Figure 3B), although this effect was less pronounced than in CD8^+^ T cells. These findings suggest that let-7 restricts CD4^+^ T cell proliferation, which, in turn, can contribute to the diminished amount of CNS-infiltrated Let-7Tg CD4^+^ T cells observed in EAE.

Given that the expansion of Let-7Tg CD4^+^ T cells was reduced, we investigated whether let-7 inhibits the expression of genes that regulate cell cycle progression and the metabolic switch in antigen-stimulated CD4^+^ T cells. Similarly to the results observed in CD8^+^ T cells, activated Let-7Tg CD4^+^ T cells expressed low levels of cyclin D2 (*Ccnd2*), cyclin-dependent kinase 6 (*Cdk6*), phosphatase Cdc25a (*Cdc25a*), and ubiquitin-conjugating enzyme Cdc34 (*Cdc34*), as well as the transcription factor Myc and several Myc target genes involved in glycolysis and protein synthesis (*Tfap4, Glut3, Hk2, Ldha, Qars, Yars*), while in Lin28Tg cells those genes were derepressed (28, 45–47) (Figures 3C,D). These results indicate that let-7 miRNAs may restrain CD4^+^ T cell proliferation by suppressing the metabolic switch and cell cycle progression. These data also suggest that Lin28Tg CD4^+^ T cells in the CNS are able to aggravate disease due to their proliferative and metabolic advantage over WT CD4^+^ T cells, despite a survival defect.

### Let-7 Negatively Regulates the Differentiation of Th17 Cells

**Figure 4:**
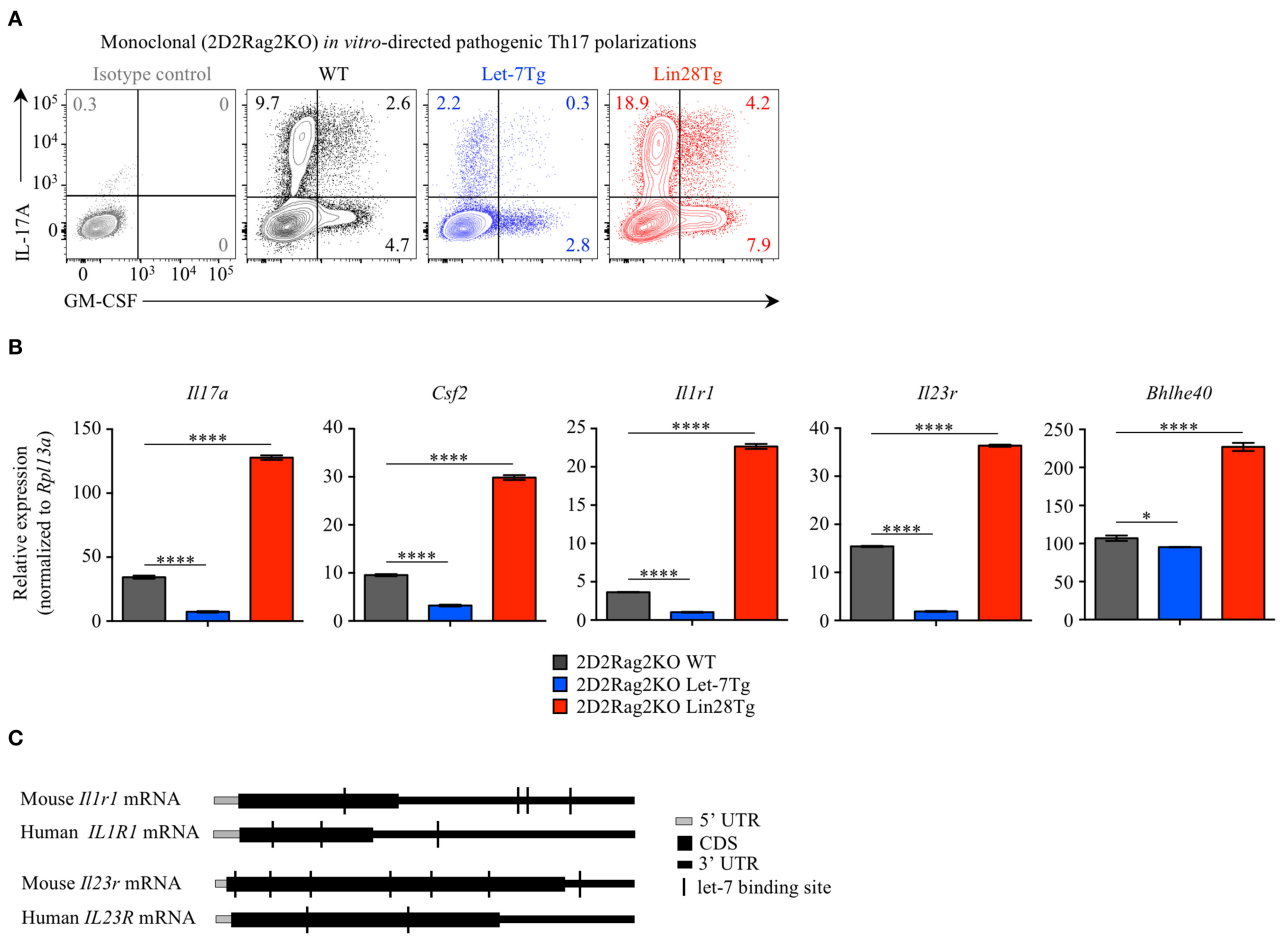
Let-7 miRNAs inhibit the acquisition of pathogenic Th17 phenotype. **(A)** Intracellular staining of CD4^+^ T cells from 2D2Rag2KO WT, 2D2Rag2KO Let-7Tg, and 2D2Rag2KO Lin28Tg mice polarized *in vitro* toward the pathogenic Th17 lineage with IL-6, IL-1β, and IL-23. Numbers indicate the frequencies of cytokine-positive cells within the indicated gates. **(B)** Quantitative RT-PCR analysis of the cytokines IL-17A (*Il17a*) and GM-CSF (*Csf2*), the cytokine receptors IL-1R1 (*Il1r1*) and IL-23R (*Il23r*), and the transcription factor Bhlhe40 (*Bhlhe40*) in *in vitro*-generated pathogenic Th17 cells from 2D2Rag2KO WT, 2D2Rag2KO Let-7Tg, and 2D2Rag2KO Lin28Tg mice from **(A)**, presented relative to results obtained for the ribosomal protein Rpl13a (control). **(C)** Diagram positioning *in silico*-identified let-7 binding sites (black vertical lines) within the mouse and human mRNA sequences of the cytokine receptors IL1-R1 (*Il1r1* and *IL1R1*, respectively) and IL-23R (*Il23r* and *IL-23R*, respectively). *p < 0.05, ****p < 0.0001 **(B)**, compared with WT using two-tailed Student's *t*-test. Data are from one experiment representative of two independent experiments **(A)** or from two independent experiments (**B**; mean ± S.E.M. of technical triplicates of each population from all mice).

Alternatively, the low frequency of effector CD4^+^ T cells in the CNS of Let-7Tg mice during EAE could be due to a defect in Th17 differentiation. Therefore, we tested the ability of let-7 miRNAs to influence the differentiation of pathogenic CD4^+^ T cells. Naïve 2D2Rag2KO CD4^+^ T cells with different levels of let-7 expression were polarized *in vitro* toward the TGFβ-independent pathogenic Th17 lineage in the presence of the cytokines IL-1β, IL-6, and IL-23 (48, 49). We confirmed that while the expression of let-7 miRNAs was downregulated over time in 2D2Rag2KO WT cells (Figure S4A), the expression of let-7g transgene remained high in 2D2Rag2KO Let-7Tg cells before and after differentiation (Figure S4B), and Lin28 expression could only be detected at significant levels in 2D2Rag2KO Lin28Tg cells (Figure S4C). Notably, very few Th17 Let-7Tg cells expressed IL-17 and GM-CSF cytokines as compared to WT controls, whereas Lin28Tg cells had an increased proportion of such cells (Figure 4A). This trend was also observed in the frequencies of IL-17^+^GM-CSF^+^ double positive cells, suggested to be the most pathogenic Th17 cells in EAE (11–15). Furthermore, the mRNA expression of *Il17a* and *Csf2* genes correlated with the frequency of IL-17A- and GM-CSF-producing cells (Figure 4B). These results suggest that let-7 expression in CD4^+^ T lymphocytes prevents the differentiation of pathogenic Th17 cells.

We noticed that the expression of the cytokine receptors *Il1r1* and *Il23r*, both previously described as essential for the differentiation of pathogenic Th17 cells in EAE (3, 5, 13, 50, 51), was profoundly inhibited in Let-7Tg and derepressed in Lin28Tg pathogenic Th17-polarized cells. Analysis of *Il1r1* and *Il23r* mRNA sequences revealed multiple potential let-7 binding motifs which can be directly targeted by let-7 (Figure 4C). In fact, such regulation was previously proposed for the mRNA of *IL23R* in human memory CD4^+^ T cells (52). Interestingly, although miRNA binding sites are most commonly found within the 3' UTRs of mRNAs, some let-7 binding sites were located within the coding sequence of mouse and human *Il1r1* and *Il23r* mRNAs. In addition, we found that the expression of the transcription factor *Bhlhe40*, shown to be induced by IL-1R signaling (9) and essential for the pathogenicity of Th17 cells in EAE (7, 8), was also compromised in Let-7Tg pathogenic Th17-polarized cells, but enhanced in Lin28Tg lymphocytes (Figure 4B). However, let-7 miRNAs had little or no impact on the expression of other genes controlling the Th17 cell fate, such as *Il6ra, Il6st, Stat3, Irf4*, and *Rorc* (Figure S4), despite the fact that *Il6ra, Stat3*, and *Rorc* are predicted direct let-7 miRNA targets (53). Thus, these results suggest that let-7 miRNAs negatively regulate the acquisition of pathogenic Th17 phenotype and may act through directly targeting *Il1r1* and *Il23r* transcripts.

Of note, in agreement with previously published reports (31–33) we confirmed that let-7 expression also blocks the differentiation of Th0, Th1, and Th2 cells (Figure S5). Interestingly, there was no obvious effect of let-7 on the generation of iTregs in polyclonal polarization cultures. However, iTreg differentiation was quite substantially inhibited by let-7 in monoclonal 2D2Rag2KO cells (Figure S5). The expression of *Ifng, Il4*, and *Csf2* mRNAs was consistent with the frequencies of IFNγ^+^, IL-4^+^ and GM-CSF^+^ cells in Th0, Th1, and Th2 cultures (Figure S6). The expression of genes encoding lineage-specific cytokine receptors, such as *Il12rb2* (Th1) and *Il4ra* (Th2), as well as lineage-specific transcription factors, including *Tbx21* (Th1) and *Gata3* (Th2), and the transcription factor *Bhlhe40*, which has been shown to promote a proinflammatory phenotype in Th1 cells (54), was also repressed in Let-7Tg Th0, Th1, and Th2 cells, but increased in Lin28Tg cells. Thus, these results suggest a broader suppressive role for let-7-miRNAs in the regulation of effector CD4^+^ T cell differentiation.

### Let-7 Prevents the Chemokine-Mediated Migration of Pathogenic Th17 Cells

**Figure 5:**
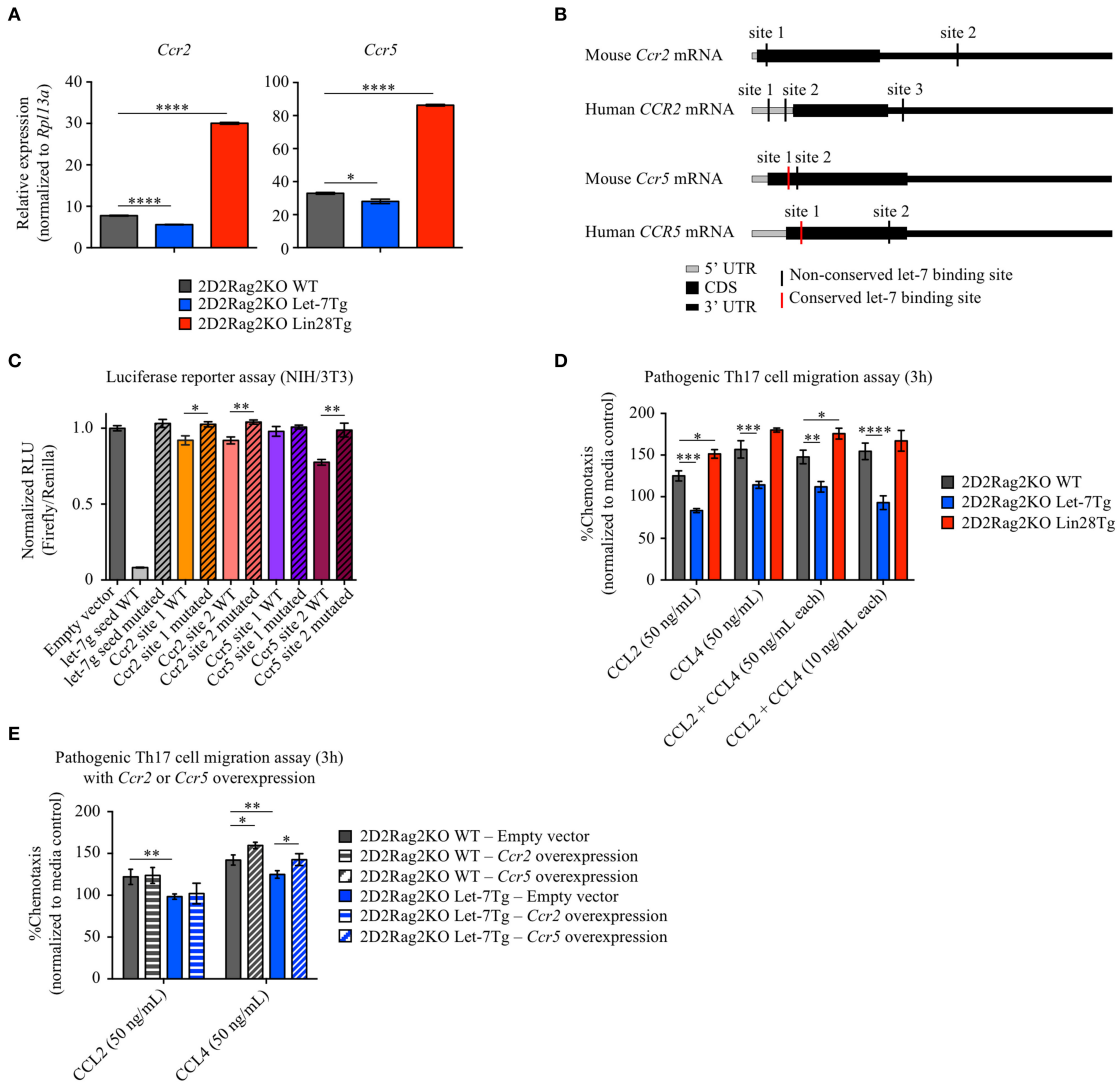
Let-7 miRNAs prevent the chemokine-dependent migration of *in vitro*-generated pathogenic Th17 cells by suppressing the expression of the chemokine receptors CCR2 and CCR5. **(A)** Quantitative RT-PCR analysis of the chemokine receptors CCR2 (*Ccr2*) and CCR5 (*Ccr5*) in *in vitro*-generated pathogenic Th17 cells from 2D2Rag2KO WT, 2D2Rag2KO Let-7Tg, and 2D2Rag2KO Lin28Tg mice, presented relative to results obtained for the ribosomal protein Rpl13a (control). **(B)** Diagram positioning *in silico*-identified conserved (red vertical lines) and non-conserved (black vertical lines) let-7 binding sites within the mouse and human mRNA sequences of the chemokine receptors CCR2 (*Ccr2* and *CCR2*, respectively) and CCR5 (*Ccr5* and *CCR5*, respectively). **(C)** Luciferase reporter assay of let-7 targeting *in-silico*-identified let-7-binding sites in mouse *Ccr2* or *Ccr5* mRNA, in NIH/3T3 cells transfected with a luciferase reporter vector containing either the wild-type or mutated variants of these binding sites, or either the wild-type or a mutated variant of the antisense seed sequence of let-7g (controls). Results are presented as relative luminescence units (RLU), calculated by normalization of Firefly luciferase activity to Renilla luciferase activity (control). **(D)** Transwell migration assay of *in vitro*-generated pathogenic Th17 cells from 2D2Rag2KO WT, 2D2Rag2KO Let-7Tg, and 2D2Rag2KO Lin28Tg mice from **(A)** in response to the chemokines CCL2 (50 ng/mL) and CCL4 (50 ng/mL) alone or in combination (50 ng/mL or 10 ng/mL each). Results are presented as percentage of cell migration in media only control, defined as 100%. **(E)** Transwell migration assay of *in vitro*-generated pathogenic Th17 cells from 2D2Rag2KO WT and 2D2Rag2KO Let-7Tg mice, transduced with empty vector (solid bars), *Ccr2*-overexpression vector (horizontally-striped bars), and *Ccr5*-overexpression vector (diagonally-striped bars) in response to the chemokines CCL2 (50 ng/mL) and CCL4 (50 ng/mL) alone. Results are presented as percentage of cell migration in media only control, defined as 100%. *p < 0.05, **p < 0.01; ***p < 0.001, ****p < 0.0001 **(A,C,D,E)**, compared with WT using two-tailed Student's *t*-test. Data are from one experiment representative of at least two independent experiments (**A,C,D,E**; mean ± S.E.M. of technical triplicates).

In addition to reduced proliferation and compromised differentiation, the lower number of effector Let-7Tg CD4^+^ T cells in the CNS could be due to impaired cell trafficking. Antigen-stimulated T cells upregulate chemokine receptors to sense, migrate, and home to the location of inflammatory sites by following gradients of chemokines (55). Two chemokine receptors, CCR2 and CCR5, have been shown to be critical for the migration of pathogenic T cells to the CNS and subsequently for EAE development (56–59). To determine whether let-7 regulates CCR2 and CCR5 expression, we measured *Ccr2* and *Ccr5* mRNA levels in *in vitro*-generated 2D2Rag2KO pathogenic Th17 cells from WT, Let-7Tg and Lin28Tg mice. Surprisingly, the expression of both *Ccr2* and *Ccr5* was very low in Let-7Tg cells, while in Lin28Tg cells it was enhanced (Figure 5A). Interestingly, we found potential let-7 binding sites within the mRNA of both *Ccr2* and *Ccr5* (Figure 5B). To test whether these binding sites were functional, we transfected NIH3T3 fibroblasts, which have high endogenous expression of let-7 miRNAs, with dual luciferase vectors containing the wild-type sequence of these binding motifs. An ability for let-7 to bind to both sites in the *Ccr2* mRNA, and one site in *Ccr5* mRNA was demonstrated by a significant reduction in luciferase activity (Figure 5C). Mutation of these binding sites restored luciferase activity, confirming direct let-7 targeting. To test whether the let-7-mediated suppression of CCR2 and CCR5 expression is sufficient to prevent chemokine-mediated migration of Th17 cells toward their specific ligands, CCL2 and CCL4, chemokine-mediated migration assays were performed. Indeed, 2D2Rag2KO Let-7Tg cells exhibited compromised trafficking in response to both chemokines alone and even in combination, while Lin28Tg cells migrated more efficiently than WT cells (Figure 5D). Although changes in cell motility can contribute to the difference in trafficking of Th17 cells, neither speed nor other intrinsic motility variables (track length, track straightness, and cell displacement) were negatively affected by the expression of let-7 (Figure S7A). To test whether let-7 miRNAs prevent the migration of pathogenic Th17 cells by inhibiting CCR2 and CCR5 expression, we overexpressed *Ccr2* or *Ccr5* in *in vitro*-generated 2D2Rag2KO WT and 2D2Rag2KO Let-7Tg pathogenic Th17 cells (Figure S7B) and subjected these cells to chemokine-mediated migration assays. Surprisingly, only overexpression of *Ccr5*, but not *Ccr2*, partially rescued the chemotaxis of 2D2Rag2KO Let-7Tg pathogenic Th17 cells toward CCL4, and enhanced the migration of 2D2Rag2KO WT pathogenic Th17 cells (Figure 5E). These results strongly suggest that let-7 miRNAs restrict the CCR5-mediated migration of pathogenic Th17 cells by directly binding to *Ccr5* mRNA and inhibiting CCR5 expression. However, additional let-7-mediated regulatory mechanisms are involved in the CCR2-mediated migration of these cells.

Altogether, our data show that let-7 miRNAs control the pathogenicity of Th17 cell in EAE by restricting their clonal expansion, inhibiting IL-1R1/IL-23R-dependent differentiation and preventing CCR2/CCR5-mediated migration to the CNS. As such, we propose that let-7 miRNAs may constitute a promising therapeutic target for the treatment of autoimmune diseases such as MS.

## Discussion

In the present study, we have identified let-7 miRNAs as critical negative regulators of pathogenic Th17 cell differentiation and EAE development. Specifically, we found that, similarly to CD8^+^ T cells (28), the expression of let-7 miRNAs in naïve CD4^+^ T cells is downregulated upon activation, which is essential for the clonal expansion, acquisition of pathogenic Th17 phenotype, and migration to the CNS. We demonstrate that high let-7 miRNA expression in activated CD4^+^ T cells confers almost complete protection against EAE by preventing CD4^+^ T cell pathogenicity and infiltration in the CNS, while in the absence of let-7 miRNAs, the development of EAE is exacerbated.

The role of miRNAs in the regulation of T cell differentiation and function has been extensively studied (60). However, despite the growing number of reports describing miRNA dysregulation in MS patients (25), their contribution to MS pathogenesis remains largely unknown. Although let-7 is one of the most highly expressed miRNA families in CD4^+^ T cells (26) and it has been shown to play regulatory roles in helper T cell responses (31–33, 35, 36), published reports have yielded conflicting conclusions regarding the contribution of let-7 miRNAs to Th17 cell pathogenicity during MS and EAE, and have remained unresolved (25). For example, Junker et al. (23) found an upregulation of let-7c in MS lesions, while Kimura et al. (36) described an increase in exosomal let-7i in MS patients and proposed a disease-promoting role for let-7i. On the other hand, Cox et al. (38) showed a downregulation of let-7d, f, i, and, together with Martinelli-Boneschi et al. (39), let-7g, in peripheral blood samples of MS patients, whereas, Guan et al. (37) reported a decrease in let-7g and let-7i in pathogenic CD4^+^ T cells in EAE, but at the same time let-7b, c, d, f and especially let-7e, were upregulated. Moreover, overexpression of let-7e in CD4^+^ T cells led to aggravated EAE, while knockdown of this miRNA attenuated the disease. Our findings are in discordance with Guan et al. (37), since we show that EAE is aggravated upon adoptive transfer of Lin28Tg naïve CD4^+^ T cells, in which all let-7 members are suppressed, into Rag2KO recipients. These disparities could be due to the use of different mouse models and treatments, as the cited study employed CD44 KO CD4^+^ T cells and lentivirus-mediated overexpression or silencing of let-7e expression, while we used transgenic mice with specific modulation of let-7 miRNA expression.

Our most striking finding is that let-7 miRNAs keep pathogenic CD4^+^ T cells from infiltrating the CNS. This effect was not due to a detrimental impact of let-7 miRNAs on the survival of activated CD4^+^ T cells, as maintenance of high let-7 miRNA expression improved the survival rate of activated CD4^+^ T cells, while let-7 deficiency caused an increase in cell death, which is consistent with our recently published observations in both naïve CD4^+^ and CD8^+^ T cells that let-7 miRNAs promote homeostatic survival through IL-7-independent stabilization of Bcl2 expression (29).

In comparison to CD8^+^ T cells (28), we observed only an incremental contribution of let-7 to the proliferation of CD4^+^ T cells, although we found let-7-mediated suppression of *Myc* and Myc target genes involved in glycolysis and protein synthesis. Furthermore, we demonstrate that let-7 miRNAs inhibit the expression of *Cdc25* and *Cdc34*, both of which are involved in the positive regulation of cell cycle progression and are also documented as direct Myc and let-7 miRNA target genes (45–47). Thus, our results indicate that let-7 miRNAs may regulate cell cycle progression in CD4^+^ T cells both directly by inhibiting *Cdc25a* and *Cdc34* and indirectly through *Myc*.

It is well-known that regulatory T cells play an indispensable role in preventing autoimmunity (61). Based on our EAE experiments, it was reasonable to hypothesize that expression of let-7 miRNAs may enhance development or function of Tregs while let-7 deficiency compromises it. Surprisingly, our data show the opposite results, in which let-7 miRNAs inhibited differentiation of monoclonal (2D2Rag2KO) Tregs *in vitro*. Furthermore, we noticed that despite the fact that Lin28Tg CD4^+^ T cells have enhanced potentials for differentiation into pathogenic Th17 cells, Lin28Tg mice are healthy and do not demonstrate signs of autoimmunity, suggesting unaltered Treg function in the absence of let-7 miRNAs. Based on these observations we can conclude that let-7 expression does not enhance differentiation or function of these cells. Therefore, in the attempt to understand the observed phenotype, we focused our research on the role let-7 miRNAs in differentiation and function of pathogenic Th17 cells.

We show that let-7 miRNAs prevent the *in vitro* differentiation of naïve CD4^+^ T cells toward the pathogenic Th17 lineage, as reflected by the reduced frequencies of IL-17A^+^, GM-CSF^+^, and IL-17A^+^GM-CSF^+^ cells, and the downregulation of the cytokine genes *Il17a* and *Csf2* (encoding GM-CSF), as well as the cytokine receptor genes *Il1r1*, and *Il23r*. Both IL-1R1 and IL-23R signaling play critical roles in Th17 cell differentiation, as mice deficient in either cytokine receptor or their respective ligand are completely resistant to EAE development (4, 50, 51, 62). It was later found that IL-23R signaling, as well as IL-1R1-mediated expression of the transcription factor Bhlhe40, induces the expression of the cytokine GM-CSF, which stimulates peripheral inflammatory macrophages and promotes their migration to the CNS, where they are responsible for demyelination and neuroaxonal damage (5, 9, 13, 14). Both Bhlhe40 and GM-CSF have been shown to be indispensable for EAE induction, since deficiency in either factor confers protection against EAE (7, 8, 10–12), and elevated GM-CSF levels have been correlated with the active phase of MS (63). In addition, two earlier reports proposed a role for specific members of the let-7 family in the regulation of *Il23r* expression. Specifically, potential let-7f binding sites were identified in *IL23R* mRNA in human memory CD4^+^ T cells (52), and loss of let-7e- and let-7f-mediated regulation of a human *IL23R* gene variant was shown to be associated with inflammatory bowel disease, due to a polymorphism in the 3' UTR of *IL-23R* mRNA sequence (64). In our study, we found additional let-7 binding sites within the mRNA sequence of *Il23r* and described the regulatory role of let-7 miRNAs in the context of pathogenic Th17 cell differentiation. Furthermore, we identified potential let-7 binding sites within the mRNA sequence of *Il1r1*, which has never been suggested to be a direct let-7 miRNA target. Therefore, we propose a novel let-7 miRNA-mediated regulatory mechanism in which let-7 miRNAs prevent both IL-1R1 and IL-23R expression in CD4^+^ T cells by directly targeting their respective transcripts. Consistent with knock-out studies, CD4^+^ T cells that do not express these cytokine receptors are unable to receive the necessary signals for the induction of Bhlhe40 and GM-CSF, thereby aborting the differentiation of encephalitogenic Th17 cells and EAE development.

We also tested whether the let-7 miRNA-mediated restriction of CNS infiltration by pathogenic CD4^+^ T cells in EAE was due to the inhibition of cell migration to the CNS. Despite no difference in intrinsic motility of *in vitro*-generated pathogenic Th17 cells, we demonstrated, using transwell assays, that let-7 repressed the chemokine-mediated migration of these cells in response to the chemokines CCL2 and CCL4 by suppressing the expression of their cognate receptors CCR2 and CCR5. Although chemokine receptors are normally upregulated in differentiating T cells, enabling them to migrate and home to the location of ongoing immune responses, CCL2 and CCL4 have been detected at high levels in the cerebrospinal fluid, brain tissue, and active lesions of patients with MS, and elevated expression of both chemokine receptors on pathogenic CD4^+^ T cells has been correlated with the active phase of MS (57, 65, 66). In fact, the role of CCR2 and CCR5 in pathogenic CD4^+^ T cell trafficking to the CNS in MS has been confirmed in EAE using receptor-deficient mice (56, 59). Although the observed dysregulation of these receptors could be indirectly resulting from the let-7-mediated inhibition of pathogenic Th17 differentiation, we found that the mRNA sequences of both chemokine receptors contain potential let-7 miRNA binding sites. We showed, using luciferase reporter assays, that both sites identified in *Ccr2* mRNA, but only one site in *Ccr5* mRNA, are functional let-7-binding sites. Furthermore, we showed, in transwell assays using *Ccr2*- and *Ccr5*-overexpressing cells, that let-7 can inhibit the CCR5-, but not the CCR2-mediated migration of pathogenic Th17 cells. Therefore, we propose that let-7 miRNAs, in addition to possibly targeting *Il1r1* and *Il23r* transcripts, also inhibit CCR5-mediated chemotaxis by directly targeting *Ccr5* mRNA in pathogenic CD4^+^ T cells. The repression of CCR2-mediated chemotaxis appears to be controlled by unknown let-7-mediated regulatory mechanism(s) and thus is not rescued by just CCR2 overexpression. In accordance with chemokine receptor deficiency studies in EAE, we can conclude that the inability to express the adequate levels of CCR5 suppresses the responsiveness of pathogenic Th17 cells to their respective ligands, thereby preventing their migration to the CNS and EAE induction.

Altogether, our data demonstrate that let-7 miRNAs have a protective effect in EAE rather than a role in promoting disease pathogenesis. Therefore, delivering let-7 miRNAs to pathogenic Th17 cells may be a promising therapeutic strategy for the treatment of MS and related autoimmune diseases.

## Materials and Methods

### Mice

C57BL/6J (WT CD45.2^+^, stock no. 000664), B6(Cg)-Rag2^tm1.1Cgn^/J (Rag2KO, stock no. 008449), B6.Cg-Col1a1^tm3(tetO−Mirlet7g/Mir21)Gqda^/J (let-7g, stock no. 023912) and B6.Cg- Gt(ROSA)26 Sor^tm1(rtTA^*^M2)Jae^/J (M2rtTA, stock no. 006965) mice were acquired from the Jackson Laboratory. let-7g and M2rtTA mice were bred to generate Let-7Tg mice. Mice with a human CD2 promoter-driven Lin28B transgene (Lin28Tg) (27) were generously provided by Alfred Singer (NCI, NIH) and C57BL/6-Tg(Tcra2D2, Tcrb2D2)1Kuch/J (2D2) mice were a kind gift from BAO. 2D2 mice were crossed on a Rag2KO background to produce 2D2Rag2KO WT mice. Let-7Tg and 2D2 mice were bred on a Rag2KO background to generate 2D2Rag2KO Let-7Tg mice. Lin28Tg and 2D2 mice were crossed on a Rag2KO background to produce 2D2Rag2KO Lin28Tg mice. Control mice used were either littermates or age and sex-matched mice. All breedings were maintained at the University of Massachusetts, Amherst. All experiments were executed according to the recommendations in the Guide for the Care and Use of Laboratory Animals of the National Institutes of Health. All mice were handled in accordance with reviewed and approved institutional animal care and use committee (IACUC) protocols (#2017-0041, #2017-0053) of the University of Massachusetts.

### Doxycycline Treatment for the Induction of Let-7 Transgene Expression

All experimental mice (including controls) were fed with 2 mg/mL doxycycline hyclate (Sigma) and 10 mg/mL sucrose in drinking water that was replaced once over the course of 4 days before the start of experiments in order for maximal let-7g expression. For EAE experiments, doxycycline treatment was maintained throughout disease course analysis, during which doxycycline-containing water was replaced every other day. For *in*-*vitro* lymphocyte cultures, lymphocyte culture media (see cell sorting and *in-vitro* culture below) was complemented with 2 μg/mL doxycycline hyclate.

### Cell Sorting and *in-vitro* Culture

Lymph nodes were collected and gently dissociated using sharp-ended forceps to release lymphocytes. Naïve CD4^+^CD44^lo^CD25^−^CD8^−^ T cells were either purified using electronic sorting after removal of B cells from whole-lymphocyte suspensions using α-mouse IgG-coated magnetic beads (BioMAg, Qiagen) or directly isolated from whole-lymphocyte suspensions using the EasySep™ Mouse Naïve CD4^+^ T Cell Isolation kit (Stem Cell Technologies) according to the manufacturer's instructions. Cells were cultured in RPMI media supplemented with 10% fetal bovine serum, 1% penicillin/streptavidin, 1% L-glutamine, 1% non-essential amino-acids, 1% sodium pyruvate, 1% HEPES and 0.3% β-mercaptoethanol. Culture media was supplemented with 2 μg/mL doxycycline, and 100 μg/mL gentamicin when necessary. Unless otherwise indicated, cells were activated with plate-bound α-CD3 (clone 2C11, 1 or 5 μg/mL) and α-CD28 (clone 37.51, 5 μg/mL).

### Induction of EAE and Disease Analysis

EAE was induced by subcutaneous immunization with the MOG_35−55_ peptide in complete Freund's adjuvant (Hooke Laboratories EK-2110) according to the manufacturer's instructions. Intraperitoneal injection of 60 ng pertussis toxin (Hooke Laboratories BT-0105) was performed 2–4 h and 26–28 h post-immunization. For adoptive-transfer experiments, intravenous injection of 2–2.5 × 10^6^ WT, Let-7Tg or Lin28Tg 2D2Rag2KO naïve CD4^+^ T cells was performed 12 h prior to immunization with MOG_35−55_. EAE symptoms were scored according to standard criteria: 0, asymptomatic; 1, limp tail; 2, hindlimb weakness; 3, hindlimb paralysis; 4, complete hindlimb and partial frontlimb paralysis; 5, moribund or death.

### Isolation of CNS-Infiltrating Cells

Experimental mice were sacrificed at the peak of EAE and perfused through the left cardiac ventricle with PBS containing 1% fetal bovine serum. Brain and spinal cord tissues were dissociated and digested with 1 mg/mL DNaseI (Roche) and 2.5 mg/mL collagenase D (Roche) for 30 min at 37°C using a gentleMACS dissociator (Miltenyi), filtered through 100-μm mesh strainers and centrifuged through a Percoll density gradient (37 and 70%). Mononuclear cells in the interphase were collected, washed twice with PBS and resuspended in lymphocyte culture media prior to *in-vitro* restimulation.

### Enzyme-Linked Immunosorbent Assay (ELISA)

Spleens from experimental mice were harvested at the peak of EAE and splenocytes were released by gentle organ dissociation using sharp-ended forceps. After erythrocyte lysis, duplicates of 2 × 10^7^ splenocytes from each mouse were restimulated in lymphocyte culture media supplemented with either 2.5, 5, or 10 μg/mL MOG_35−55_ (Hooke Laboratories DS-0111) in the presence of 2 μg/mL doxycycline hyclate. Cytokine concentrations were measured in supernatants collected from restimulated cells after 5 days in culture. Concentrations of secreted IL-17, GM-CSF, and IFNγ were measured using matching capture and biotinylated detection mAbs (BD Pharmingen) in a sandwich ELISA. HRP-conjugated streptavidin and HRP substrate from the TMB ELISA kit (Pierce) were applied for the quantification of HRP activity at 450 nm using a Synergy™ 2 Multi-Mode Microplate Reader (Biotek).

### CTV and CFSE Labeling

Naïve CD4^+^ T cells were labeled at 1 × 10^6^ cells/mL in PBS containing 2.5 μM CTV or 1 μM CFSE, both obtained from Invitrogen, for 15 min at 37°C. The labeling reaction was stopped by washing the cells with lymphocyte culture media prior to use in experiments.

### *In-vitro* Proliferation Assay

CTV-labeled WT, Let-7Tg, and Lin28Tg cells were activated with plate-bound α-CD3 (clone 2C11, 5 μg/mL) and α-CD28 (clone 37.51, 5 μg/mL). Cells were cultured for 3 days prior to CTV dilution profile analysis by flow cytometry.

### *In-vitro* Differentiation of CD4^+^ T Cells

Naïve CD4^+^ T cells (1 × 10^6^) were activated with soluble α-CD3 (clone 2C11, 2 μg/mL) in the presence of irradiated WT splenocytes (5 × 10^6^) and cultured for 5 days in lymphocyte culture media. In some experiments, whole-splenocyte suspensions were depleted of CD4^+^ and CD8^+^ T cells using α-mouse CD4 (clone L3T4) and α-mouse CD8 (clone Ly-2) microbeads (Miltenyi) followed by magnetic-activated cell sorting. For pathogenic Th17 differentiation, culture media was further supplemented with 20 ng/mL IL-6 (Miltneyi), 10 ng/mL IL-1β (Miltenyi), 10 ng/mL IL-23 (R&D Systems), 10 μg α-IFNγ mAbs (clone XMG1.2, BioXCell) and 10 μg/mL α-IL-4 mAbs (clone 11B11, BioXCell). For Th0 differentiation, culture media was further supplemented with 200 U/mL IL-2 (Peprotech). For Th1 differentiation, culture media was further supplemented with 200 U/mL IL-2, 10 ng/mL IL-12 (Peprotech) and 10 μg/mL α-IL-4 mAbs (clone 11B11, BioXCell). For Th2 differentiation, culture media was further supplemented with 200 U/mL IL-2, 10 ng/mL IL-4 (Peprotech) and 10 μg/mL α-IFNγ mAbs (clone XMG1.2, BioXCell). For iTreg differentiation, naïve CD4^+^ T cells were stimulated with 10 μg/mL soluble α-CD3 (clone 2C11, BD Pharmingen) and culture media was further supplemented with 100 U/mL IL-2 and 5 ng/mL TGF-β.

### *Ccr2* and *Ccr5* Overexpression and Retroviral Transduction

The *Ccr2* open reading frame (ORF) was obtained from GenScript (plasmid ID OMu22965D) and *Ccr5* cDNA was obtained from Dharmacon (clone ID 12774). *Ccr2* and *Ccr5* ORFs were cloned into the pMRX-IRES-GFP plasmid, containing a green fluorescent protein (GFP) reporter (67). Empty pMRX-IRES-GFP plasmids were used as controls. Retrovirus supernatants were produced by transfecting Platinum-E (Plate-E) retroviral packaging cells (68) using Transporter 5 transfection reagent (Polysciences). Retrovirus supernatants were concentrated 10x in lymphocyte culture media with PEG-it™ virus concentration reagent (System Biosciences) prior to cell transduction. 2D2Rag2KO CD4^+^ T cells were retrovirally transduced 24 h after activation with 10x-concentrated retrovirus supernatants by spin-infection (660 × g for 90 min at 37°C) in the presence of polybrene (4 μg/mL). Transduction media was replaced with pathogenic Th17-polarizing lymphocyte culture media 4 h after spin-infection. Analysis of transduced cells was performed by gating on the GFP^+^ cell population.

### Flow Cytometry

For analysis of surface markers, live cells were treated with α-CD16/32 Fc block (2.4G2, BD Pharmingen, RRID:AB_394657) prior to staining with antibodies against surface markers for 30 min at 4°C. For intracellular cytokine staining, cell suspensions were restimulated *in vitro* for a total of 4 h with 50 ng/mL phorbol 12-myristate 13-acetate (PMA, Sigma) and 1 μM Ionomycin (Sigma) with addition of 2 μM monensin (eBioscience) in the last 2 h of restimulation to inhibit secretion. After surface marker staining, cells were stained with the Live/Dead fixable Aqua Dead Cell Stain Kit (Thermo Fisher Scientific) according to the manufacturer's instructions. Before intracellular staining, cells were fixed and permeabilized for 30 min at 4°C using the Cytofix/Cytoperm solution kit (BD Biosciences) for cytokine staining or the Foxp3/ Transcription factor staining buffer set (eBioscience) for transcription factor staining according to the manufacturer's instructions. Samples were acquired on a BD LSR Fortessa flow cytometer (BD Biosciences) and data analysis was performed using FlowJo software (TreeStar).

### Antibodies

The following monoclonal antibodies were used for flow cytometry: CD4 (RM4-5, Biolegend, RRID:AB_493374), CD25 (PC61, Biolegend, RRID:AB_312860), FOXP3 (FJK-16S, eBioscience, RRID:AB_465935), GM-CSF (MP1-22E9, Biolegend, RRID:AB_315381), IFNγ (XMG1.2, Biolegend, RRID:AB_315399), IL-4 (11B11, BD Pharmingen, RRID:AB_395391), IL-17A (17B7, eBioscience, RRID:AB_763580).

### RNA Isolation and Quantitative RT-PCR

RNA was isolated using the QIAGEN miRNeasy (QIAGEN) or the Total RNA Purification kit (Norgen Biotek) according to the manufacturer's instructions. Genomic DNA was eliminated using the DNA-free DNA removal kit (Invitrogen). cDNA of mRNA-encoded genes was synthesized using the SuperScript III Reverse Transcriptase kit (Invitrogen) or the SensiFast™ cDNA synthesis kit (Bioline). cDNA of miRNAs was synthesized using the TaqMan MicroRNA Reverse Transcription kit (Applied Biosystems). SYBR Green or TaqMan quantitative RT-PCR were executed using the SensiFast™SYBR Lo-Rox kit (Bioline) or the SensiFast™ Probe Lo-Rox kit (Bioline), respectively. The list of specific SYBR Green amplification primers (Integrated DNA Technologies), TaqMan gene (Integrated DNA Technologies or Thermo Fisher Scientific) and TaqMan microRNA assays (Thermo Fisher Scientific) used can be found in Table S1. Quantitative RT-PCR data was acquired using a QuantStudio 6 Flex Real-Time PCR system and analyzed using QuantStudio Real-Time PCR software (Applied Biosystems).

### *In-silico* Prediction of Let-7 Binding Sites

Let-7 binding sites were identified by searching for complete or partial continuous matches to the extended let-7 seed sequence “TACTACCTCA” in the complete mRNA sequences of the indicated mouse and human genes, and are available in Table S2. A 6 bp-long perfect match was considered as minimum requirement for a potential binding site. Conservation was assessed according to the retention of the binding site position within corresponding mouse and human mRNA sequences upon optimal GLOBAL pairwise alignment using BioEdit software (Tom Hall, Ibis Therapeutics).

### Luciferase Reporter Assays

NIH/3T3 cells (ATCC) were transfected with the pmirGLO vector (Promega) containing either the wild-type *in-silico* predicted let-7-binding sites within *Ccr2* and *Ccr5* mouse mRNA, or mutated variants of these binding sites, or either the wild-type or a mutated variant of the antisense seed sequence of let-7g, using Lipofectamine and Plus Reagent (Invitrogen). Firefly luciferase activity was measured 48 h post-transfection and was normalized to Renilla luciferase activity, using the Dual-Luciferase Assay Reporter kit (Promega), on a POLARstar Omega 96-well plate reader (BMG Labtech).

### Motility in Collagen Matrices

*In vitro*-differentiated pathogenic Th17 cells from 2D2Rag2KO WT, 2D2Rag2KO Let-7Tg, and 2D2Rag2KO Lin28Tg mice were harvested at day 5, labeled with CFSE, and resuspended in RPMI/10% FBS. PureCol EZ Gel (Advanced BioMatrix) was added to cells in RPMI/10% FBS to obtain a final collagen gel concentration of 1.6 mg/mL with a final cell concentration of 1.25 × 10^6^ cells/mL. Collagen gels were allowed to fully polymerize for 1 h at 37°C prior to imaging the cells for 20 min at 10-s intervals with a modified inverted epi-fluorescence microscope (Axio Observer.Z1, Carl Zeiss). Data was analyzed using Imaris software (Bitplane).

### Transwell Assay

*In vitro*-differentiated pathogenic Th17 cells from 2D2Rag2KO WT, 2D2Rag2KO Let-7Tg, and 2D2Rag2KO Lin28Tg mice were harvested at day 5, washed in RPMI/10% FBS and resuspended at 5x10^6^ cells/mL in RPMI/10% FBS. Chemotaxis toward 600 μL control media, Ccl2 and Ccl4 alone (50 ng/mL) or in combination (50 ng/mL or 10 ng/mL each) in the lower chamber of a 24-well plate was assessed by incubating 100 μL cell suspension in the upper chamber of 24-well 6.5 mm transwell inserts with a 5-μm pore polycarbonate membrane (Corning) at 37°C for 3 h. Percent chemotaxis was measured by manually counting the number of cells present in the lower chamber and normalized to cell counts obtained in control media for each condition.

### Statistics

Data statistical analysis was performed with Prism 7 (GraphPad software) or RStudio software (RStudio Team). *P*-values were determined using a two-tailed Student's *t*-test or a two-way ANOVA, as indicated on the figure legends. A *p*-value < 0.05 was considered significant (*p < 0.05, **p < 0.01, ***p < 0.001, ****p < 0.0001).

## Data Availability Statement

The raw data supporting the conclusions of this manuscript will be made available by the authors, without undue reservation, to any qualified researcher.

## Ethics Statement

This study was carried out in accordance with the guidelines of the US National Institutes of Health and the protocols were approved by the IACUC of the University of Massachusetts.

## Author Contributions

CA, EP, and LP conceptualized and designed the study, performed experiments, and interpreted the results. JV, CD, VL, and MK performed experiments. RL, EI, and SP provided technical support. CA drafted the manuscript. CA, AW, VL, MK, LM, BO, EP, and LP critically revised the manuscript.

### Conflict of Interest

The authors declare that the research was conducted in the absence of any commercial or financial relationships that could be construed as a potential conflict of interest.

## Supplementary Material

Figure S1 Let-7 miRNAs are highly expressed in naïve CD4^+^ T cells, but get downregulated upon activation, proportionally to the duration and strength of TCR stimulation. **(A)** Quantitative RT-PCR analysis of individual let-7 miRNA expression in naïve CD4^+^ T cells activated with plate-bound α-CD3 mAbs (5 μg/mL) and α-CD28 mAbs (5 μg/mL) for increasing time periods as indicated, presented relative to results obtained for the small nuclear RNA U6 (control) and normalized to the unstimulated (0 h) control. **(B)** Quantitative RT-PCR analysis of individual let-7 miRNA expression in naïve CD4^+^ T cells activated for 24 h with plate-bound α-TCR mAbs (as indicated) and α-CD28 mAbs (5 μg/mL), presented relative to results obtained for the small nuclear RNA U6 (control) and normalized to the unstimulated (0 h) control. **(C)** ELISA analysis of IL-17, IFNγ, and GM-CSF concentration in the supernatants of splenocytes from vehicle- (no dox) or doxycycline- (+ dox) treated WT vs. Let-7Tg mice harvested at the peak of disease (day 9–15 post-immunization) and restimulated for 5 days *in vitro* with 20 μg/mL MOG_35−55_. ^*^*p* < 0.05, ^***^*p* < 0.001, ^****^*p* < 0.0001 **(C)**, compared with WT using two-tailed Student's *t*-test **(C)**. Data are from one experiment representative of two independent experiments (**A,B**; mean ± S.E.M. of technical triplicates) or from one experiment (**C**; mean ± S.E.M. of technical triplicates of each population from all mice).

Figure S2 Let-7 miRNAs control the development of active EAE by negatively regulating the pathogenicity of monoclonal CD4^+^ T cells. **(A)** Mean clinical scores in 2D2Rag2KO WT (*n* = 4), 2D2Rag2KO Let-7Tg (*n* = 5) and 2D2Rag2KO Lin28Tg (*n* = 3) mice immunized with MOG_35−55_ in complete Freund's adjuvant (CFA) and pertussis toxin (60 ng). **(B)** Number of total mononuclear cells at the peak of the disease (day 9 post-immunization) in the CNS of 2D2Rag2KO WT, 2D2Rag2KO Let-7Tg, and 2D2Rag2KO Lin28Tg mice. **(C)** Number of CNS-infiltrated CD4^+^ T cells at the peak of the disease (day 9–15 post-immunization) in 2D2Rag2KO WT, 2D2Rag2KO Let-7Tg, and 2D2Rag2KO Lin28Tg mice as analyzed by flow cytometry. **(D)** Intracellular staining of CD4^+^ T cells from the CNS of 2D2Rag2KO WT, 2D2Rag2KO Let-7Tg, and 2D2Rag2KO Lin28Tg mice (left). Numbers indicate the frequencies of cytokine-positive cells within the indicated gates. ^*^*p* < 0.05, ^**^*p* < 0.01; ^****^*p* < 0.0001 **(A–C)**, compared with WT employing two-way ANOVA **(A)** or using two-tailed Student's *t*-test **(B,C)**. Data are from two combined independent experiments (**A–C**; mean ± S.E.M. of each population from all mice) or one experiment representative of two independent experiments **(D)**.

Figure S3 Let-7 TG pathogenic CD4^+^ T cells can be found in the spleen of EAE-induced mice. Quantification of total cell numbers and CD4^+^ T cell numbers in the spleens of Rag2KO recipient mice that received 2D2Rag2KO WT, 2D2Rag2KO Let-7Tg, and 2D2Rag2KO Lin28Tg cells. ^*^*p* < 0.05 compared with WT employing two-tailed Student's *t*-test. Data are from one experiment (mean ± S.E.M. of each population from all mice).

Figure S4 let-7 miRNAs also get downregulated over time during pathogenic Th17 differentiation, and do not prevent the expression of several genes that regulate Th17 cell differentiation. **(A)** Quantitative RT-PCR analysis of individual let-7 miRNA expression in naive 2D2Rag2KO WT CD4^+^ T cells and during *in-vitro*-generation of 2D2RagKO WT pathogenic Th17 cells at the indicated time points, presented relative to results obtained for the small nuclear RNA U6 (control), and normalized to results obtained for naive 2D2Rag2KO WT CD4^+^ T cells. **(B)** Quantitative RT-PCR analysis of individual let-7 miRNA expression in naive CD4^+^ T cells and day-5 *in-vitro*-generated pathogenic Th17 cells from 2D2Rag2KO WT, 2D2Rag2KO Let-7Tg, and 2D2Rag2KO Lin28Tg mice, presented relative to results obtained for the small nuclear RNA U6 (control), and normalized to results obtained for naive 2D2Rag2KO WT CD4^+^ T cells. **(C)** Quantitative RT-PCR analysis of *Lin28a* and *Lin28b* in naive 2D2Rag2KO WT and 2D2Rag2KO Lin28Tg CD4^+^ T cells, as well as during *in-vitro*-generation of 2D2RagKO WT pathogenic Th17 cells at the indicated time points presented relative to results obtained for the small nuclear RNA U6 (control), and normalized to results obtained for naive 2D2Rag2KO WT CD4^+^ T cells. **(D)** Quantitative RT-PCR analysis of the IL-6 cytokine receptor components IL-6Rα (*Il6ra*) and IL-6ST (*Il6st*), and the transcription factors STAT3 (*Stat3*), IRF4 (*Irf4*), and RORγt (*Rorc*) in *in vitro*-generated pathogenic Th17 cells from 2D2Rag2KO WT, 2D2Rag2KO Let-7Tg, and 2D2Rag2KO Lin28Tg mice. ^*^*p* < 0.05, ^***^*p* < 0.001, ^****^*p* < 0.0001, compared with WT using two-tailed Student's *t*-test **(D)**. Data are from one experiment representative of at least two experiments (**A–D**; mean ± S.E.M. of technical replicates).

Figure S5 let-7 miRNAs inhibit the differentiation of several helper T cell subsets generated from both polyclonal and monoclonal naïve CD4^+^ T cells *in vitro*. Intracellular staining of CD4^+^ T cells from polyclonal **(A)** or monoclonal 2D2Rag2KO **(B)** WT, Let-7Tg and Lin28Tg mice polarized *in vitro* toward the Th0, Th1, Th2, and iTreg lineages. Numbers indicate the frequencies of cytokine-positive cells within the indicated gates. Data are from one experiment representative of seven **(A)** or six **(B)** independent experiments.

Figure S6 let-7 miRNAs negatively regulate the expression of genes controlling the differentiation of Th0, Th1, and Th2 cells generated *in vitro*. Quantitative RT-PCR analysis of the cytokines IFNγ (*Ifng*), GM-CSF (*Csf2*), and IL-4 (*Il4*), the cytokine receptors IL12Rβ2 (*Il12rb2*) and IL4Rα (*Il4ra*), as well as the transcription factors T-bet (*Tbx21*), Bhlhe40 (*Bhlhe40*), and GATA3 (*Gata3*) in naïve CD4^+^ T cells and *in vitro*-generated Th0, Th1, and Th2 cells from WT, Let-7Tg, and Lin28Tg mice, presented relative to results obtained for the ribosomal protein Rpl13a (control). ^*^*p* < 0.05, ^**^*p* < 0.01; ^***^*p* < 0.001, ^****^*p* < 0.0001 compared with WT using two-tailed Student's *t*-test. Data are from one experiment representative of two independent experiments (mean ± S.E.M. of technical triplicates of each population from all mice).

Figure S7 let-7 miRNAs do not control the intrinsic motility of *in vitro*-generated pathogenic Th17 cells. **(A)** Speed, track length, track straightness, and displacement of migrating CFSE-labeled *in vitro*-generated pathogenic Th17 cells from 2D2Rag2KO WT, 2D2Rag2KO Let-7Tg, and 2D2Rag2KO Lin28Tg mice embedded in collagen matrices. **(B)** Quantitative RT-PCR analysis of the chemokine receptors CCR2 (*Ccr2*) and CCR5 (*Ccr5*) in *in vitro*-generated pathogenic Th17 cells from 2D2Rag2KO WT and 2D2Rag2KO Let-7Tg mice, transduced with empty vector (solid bars), *Ccr2*-overexpression vector (horizontally-striped bars), and *Ccr5*-overexpression vector (diagonally-striped bars), presented relative to results obtained for the ribosomal protein Rpl13a (control). ^*^*p* < 0.05, ^**^*p* < 0.01, ^****^*p* < 0.0001, compared with WT using two-tailed Student's *t*-test. Data are from one experiment representative of two experiments (**B**, mean ± S.E.M. of technical triplicates of each population from all mice), or from one experiment (**A**, mean ± S.E.M. of at least 175 analyzed cells of each population from all mice).

Table S1 List of all qPCR primers and probes used.

Table S2 List of let-7-binding sites identified in mouse and human Ccr2 and Ccr5 mRNA sequences.
