# Supplementary figures and images for: Differentiation of Pathogenic Th17 Cells Is Negatively Regulated by Let-7 MicroRNAs in a Mouse Model of Multiple Sclerosis
Source: Front Immunol. 2020 Jan 17;10:3125. doi: 10.3389/fimmu.2019.03125 (PMC6978752; doi:10.3389/fimmu.2019.03125)

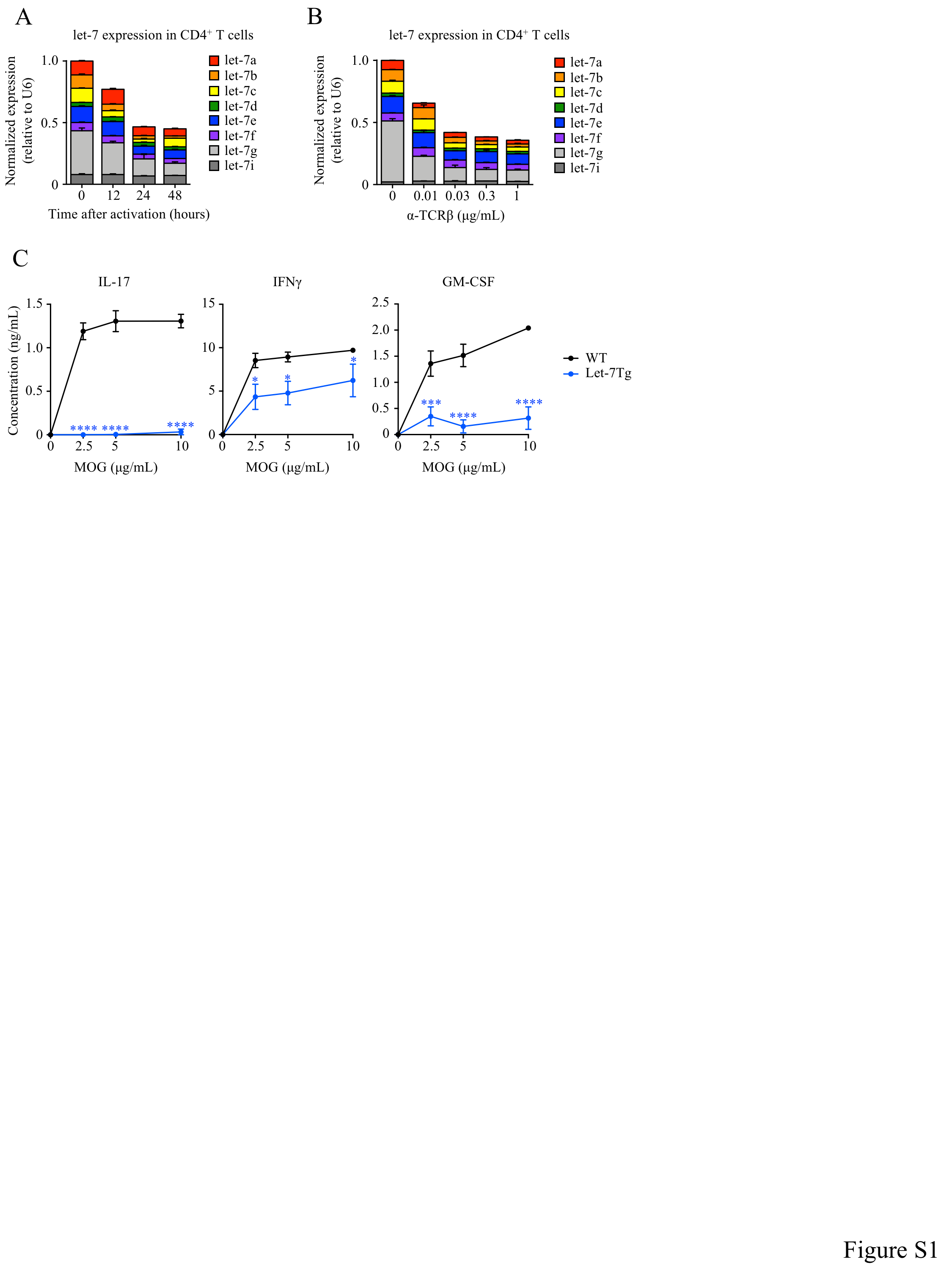

Supplement: Figure S1 — Let-7 miRNAs are highly expressed in naïve CD4+ T cells, but get downregulated upon activation, proportionally to the duration and strength of TCR stimulation. (A) Quantitative RT-PCR analysis of individual let-7 miRNA expression in naïve CD4+ T cells activated with plate-bound α-CD3 mAbs (5 μg/mL) and α-CD28 mAbs (5 μg/mL) for increasing time periods as indicated, presented relative to results obtained for the small nuclear RNA U6 (control) and normalized to the unstimulated (0 h) control. (B) Quantitative RT-PCR analysis of individual let-7 miRNA expression in naïve CD4+ T cells activated for 24 h with plate-bound α-TCR mAbs (as indicated) and α-CD28 mAbs (5 μg/mL), presented relative to results obtained for the small nuclear RNA U6 (control) and normalized to the unstimulated (0 h) control. (C) ELISA analysis of IL-17, IFNγ, and GM-CSF concentration in the supernatants of splenocytes from vehicle- (no dox) or doxycycline- (+ dox) treated WT vs. Let-7Tg mice harvested at the peak of disease (day 9–15 post-immunization) and restimulated for 5 days in vitro with 20 μg/mL MOG35−55. *p < 0.05, ***p < 0.001, ****p < 0.0001 (C), compared with WT using two-tailed Student's t-test (C). Data are from one experiment representative of two independent experiments (A,B; mean ± S.E.M. of technical triplicates) or from one experiment (C; mean ± S.E.M. of technical triplicates of each population from all mice). [file Image_1.TIFF]

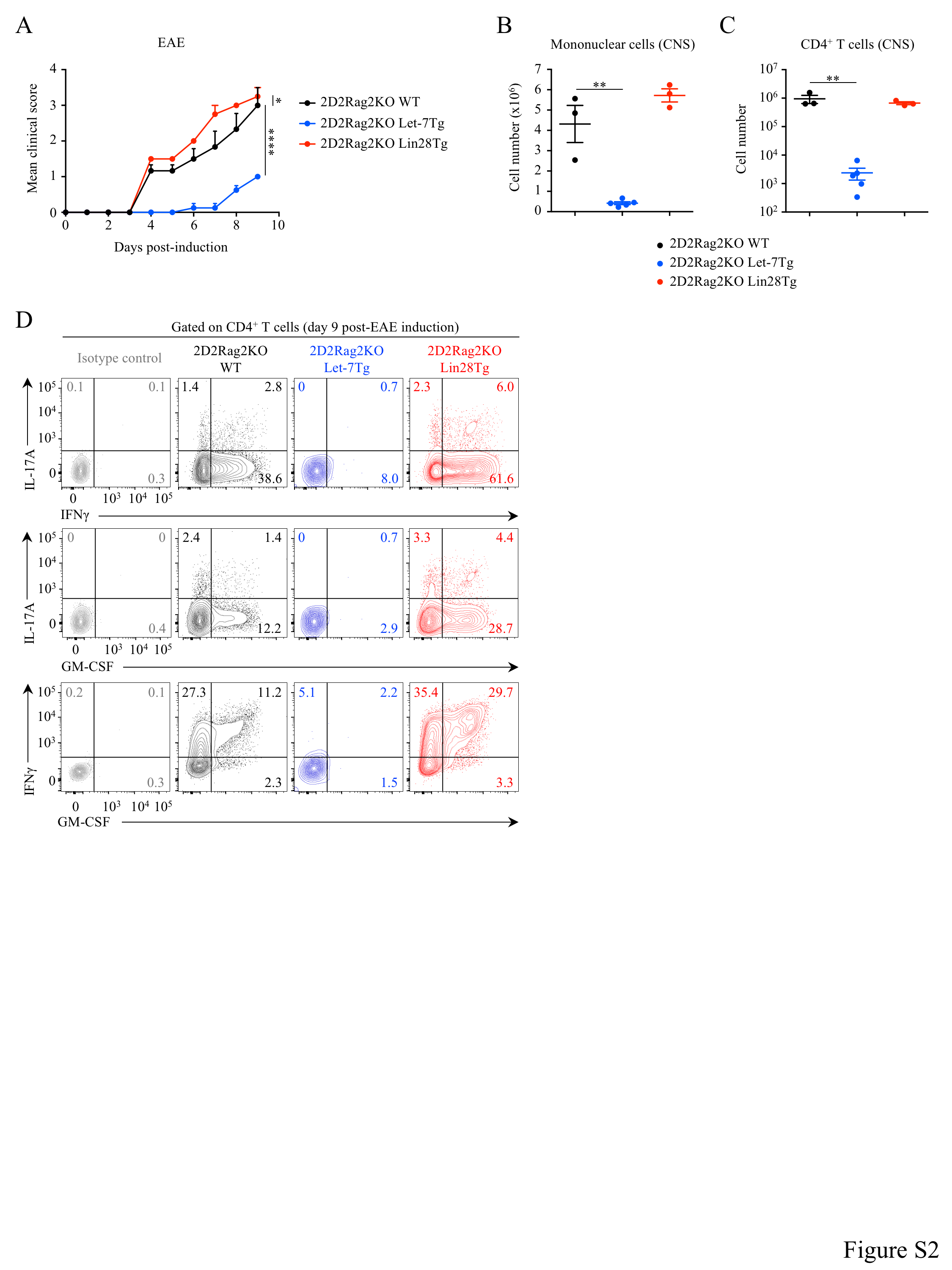

Supplement: Figure S2 — Let-7 miRNAs control the development of active EAE by negatively regulating the pathogenicity of monoclonal CD4+ T cells. (A) Mean clinical scores in 2D2Rag2KO WT (n = 4), 2D2Rag2KO Let-7Tg (n = 5) and 2D2Rag2KO Lin28Tg (n = 3) mice immunized with MOG35−55 in complete Freund's adjuvant (CFA) and pertussis toxin (60 ng). (B) Number of total mononuclear cells at the peak of the disease (day 9 post-immunization) in the CNS of 2D2Rag2KO WT, 2D2Rag2KO Let-7Tg, and 2D2Rag2KO Lin28Tg mice. (C) Number of CNS-infiltrated CD4+ T cells at the peak of the disease (day 9–15 post-immunization) in 2D2Rag2KO WT, 2D2Rag2KO Let-7Tg, and 2D2Rag2KO Lin28Tg mice as analyzed by flow cytometry. (D) Intracellular staining of CD4+ T cells from the CNS of 2D2Rag2KO WT, 2D2Rag2KO Let-7Tg, and 2D2Rag2KO Lin28Tg mice (left). Numbers indicate the frequencies of cytokine-positive cells within the indicated gates. *p < 0.05, **p < 0.01; ****p < 0.0001 (A–C), compared with WT employing two-way ANOVA (A) or using two-tailed Student's t-test (B,C). Data are from two combined independent experiments (A–C; mean ± S.E.M. of each population from all mice) or one experiment representative of two independent experiments (D). [file Image_2.TIFF]

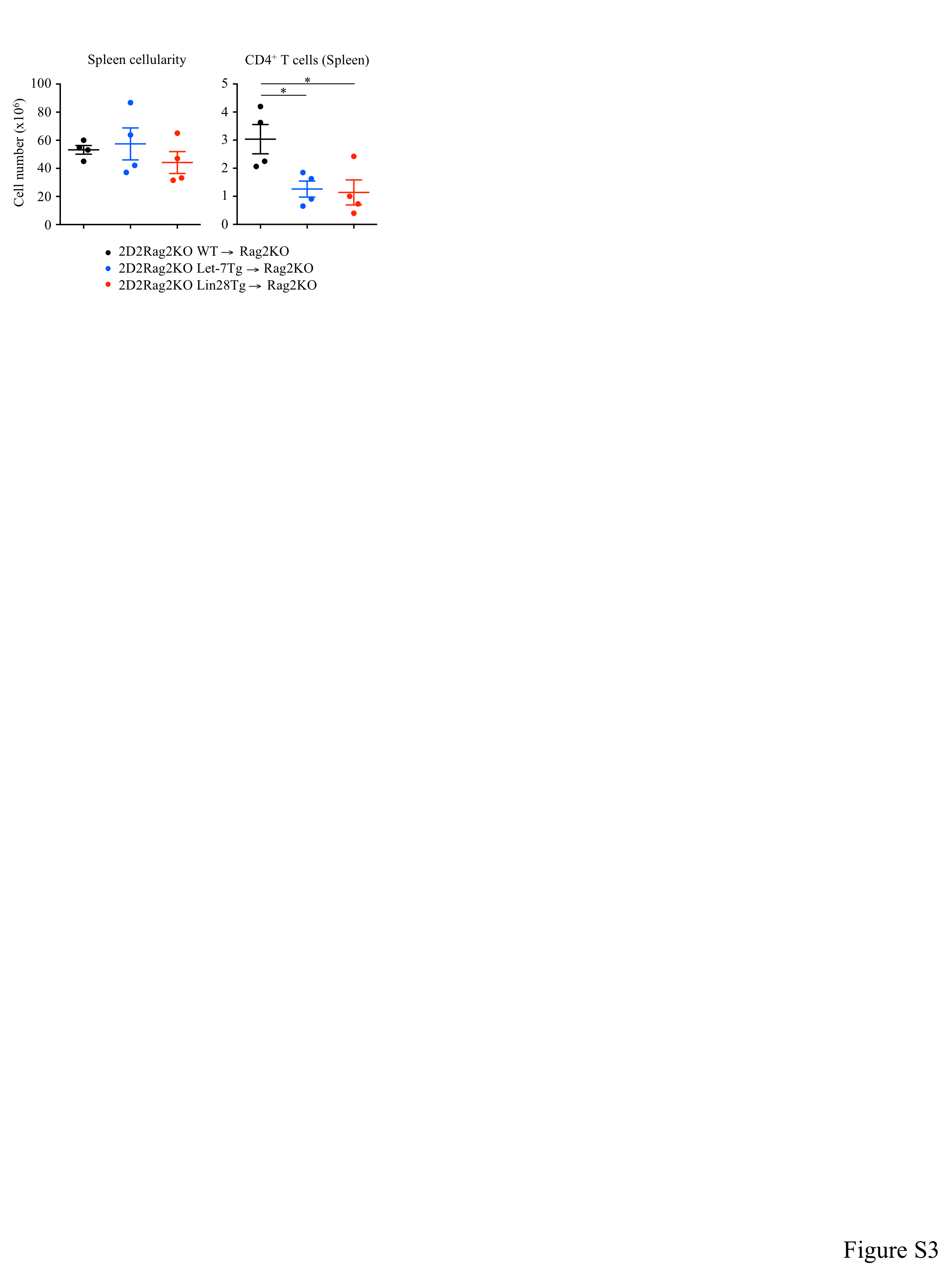

Supplement: Figure S3 — Let-7 TG pathogenic CD4+ T cells can be found in the spleen of EAE-induced mice. Quantification of total cell numbers and CD4+ T cell numbers in the spleens of Rag2KO recipient mice that received 2D2Rag2KO WT, 2D2Rag2KO Let-7Tg, and 2D2Rag2KO Lin28Tg cells. *p < 0.05 compared with WT employing two-tailed Student's t-test. Data are from one experiment (mean ± S.E.M. of each population from all mice). [file Image_3.TIFF]

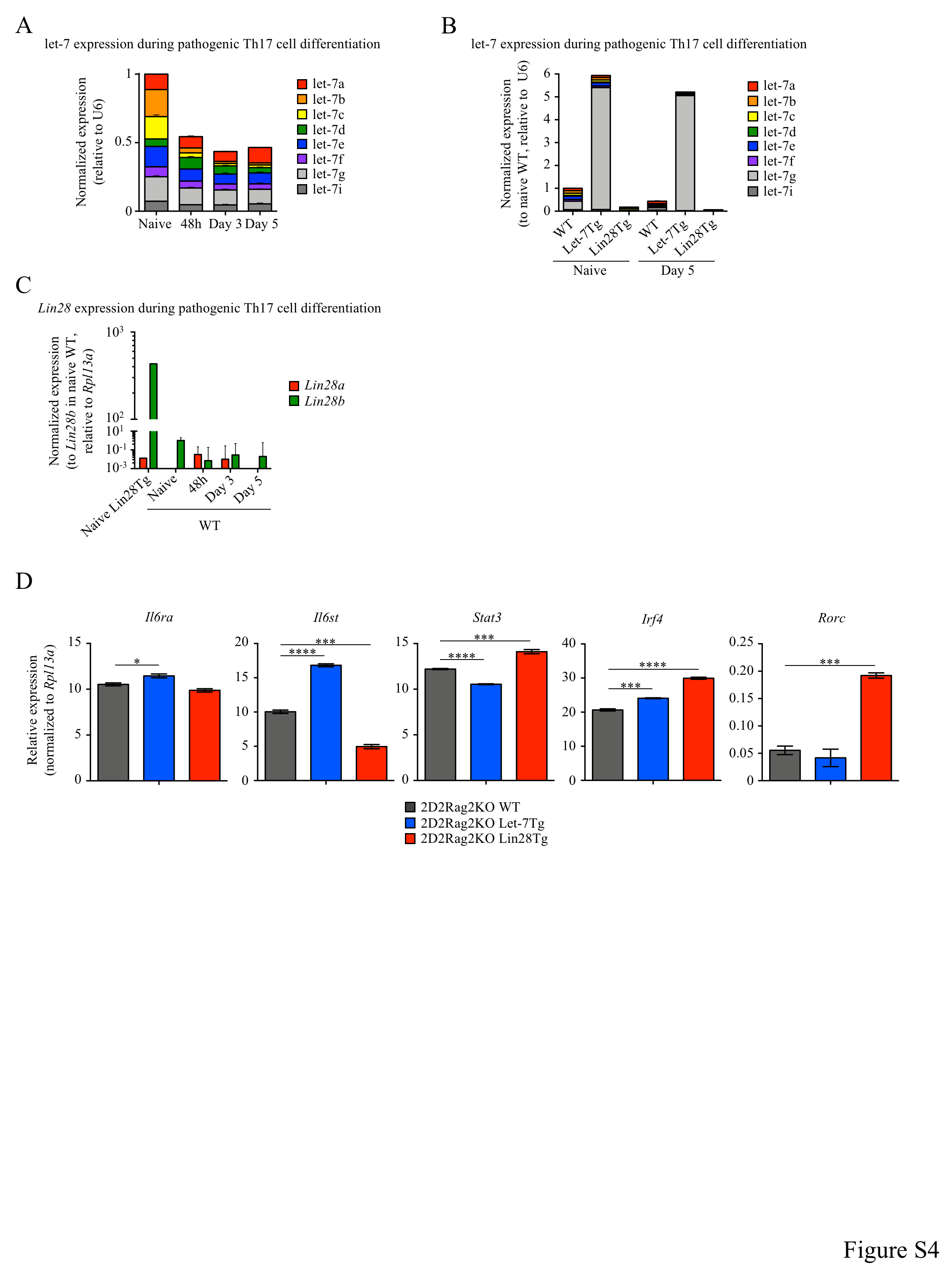

Supplement: Figure S4 — let-7 miRNAs also get downregulated over time during pathogenic Th17 differentiation, and do not prevent the expression of several genes that regulate Th17 cell differentiation. (A) Quantitative RT-PCR analysis of individual let-7 miRNA expression in naive 2D2Rag2KO WT CD4+ T cells and during in-vitro-generation of 2D2RagKO WT pathogenic Th17 cells at the indicated time points, presented relative to results obtained for the small nuclear RNA U6 (control), and normalized to results obtained for naive 2D2Rag2KO WT CD4+ T cells. (B) Quantitative RT-PCR analysis of individual let-7 miRNA expression in naive CD4+ T cells and day-5 in-vitro-generated pathogenic Th17 cells from 2D2Rag2KO WT, 2D2Rag2KO Let-7Tg, and 2D2Rag2KO Lin28Tg mice, presented relative to results obtained for the small nuclear RNA U6 (control), and normalized to results obtained for naive 2D2Rag2KO WT CD4+ T cells. (C) Quantitative RT-PCR analysis of Lin28a and Lin28b in naive 2D2Rag2KO WT and 2D2Rag2KO Lin28Tg CD4+ T cells, as well as during in-vitro-generation of 2D2RagKO WT pathogenic Th17 cells at the indicated time points presented relative to results obtained for the small nuclear RNA U6 (control), and normalized to results obtained for naive 2D2Rag2KO WT CD4+ T cells. (D) Quantitative RT-PCR analysis of the IL-6 cytokine receptor components IL-6Rα (Il6ra) and IL-6ST (Il6st), and the transcription factors STAT3 (Stat3), IRF4 (Irf4), and RORγt (Rorc) in in vitro-generated pathogenic Th17 cells from 2D2Rag2KO WT, 2D2Rag2KO Let-7Tg, and 2D2Rag2KO Lin28Tg mice. *p < 0.05, ***p < 0.001, ****p < 0.0001, compared with WT using two-tailed Student's t-test (D). Data are from one experiment representative of at least two experiments (A–D; mean ± S.E.M. of technical replicates). [file Image_4.TIFF]

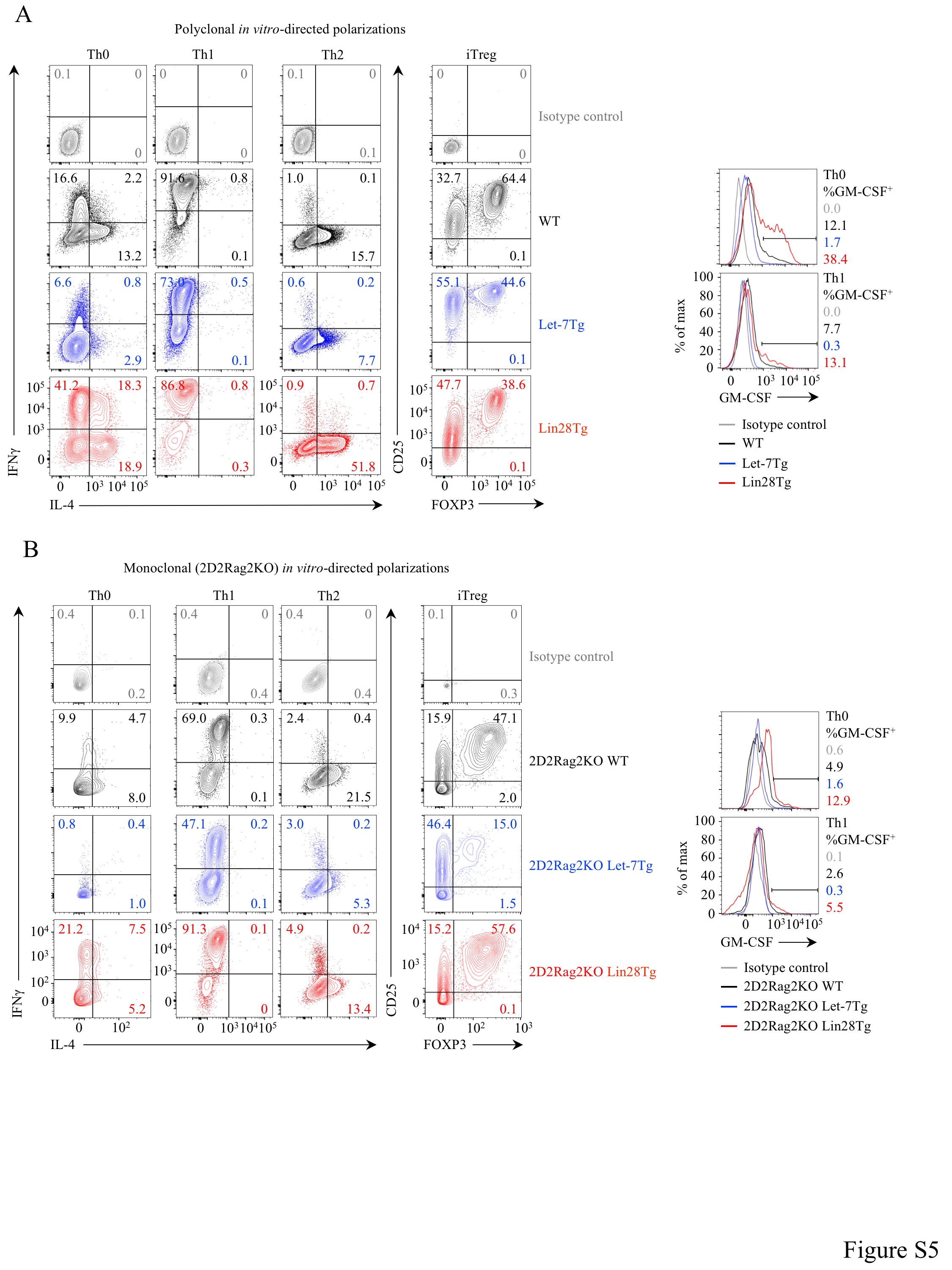

Supplement: Figure S5 — let-7 miRNAs inhibit the differentiation of several helper T cell subsets generated from both polyclonal and monoclonal naïve CD4+ T cells in vitro. Intracellular staining of CD4+ T cells from polyclonal (A) or monoclonal 2D2Rag2KO (B) WT, Let-7Tg and Lin28Tg mice polarized in vitro toward the Th0, Th1, Th2, and iTreg lineages. Numbers indicate the frequencies of cytokine-positive cells within the indicated gates. Data are from one experiment representative of seven (A) or six (B) independent experiments. [file Image_5.TIFF]

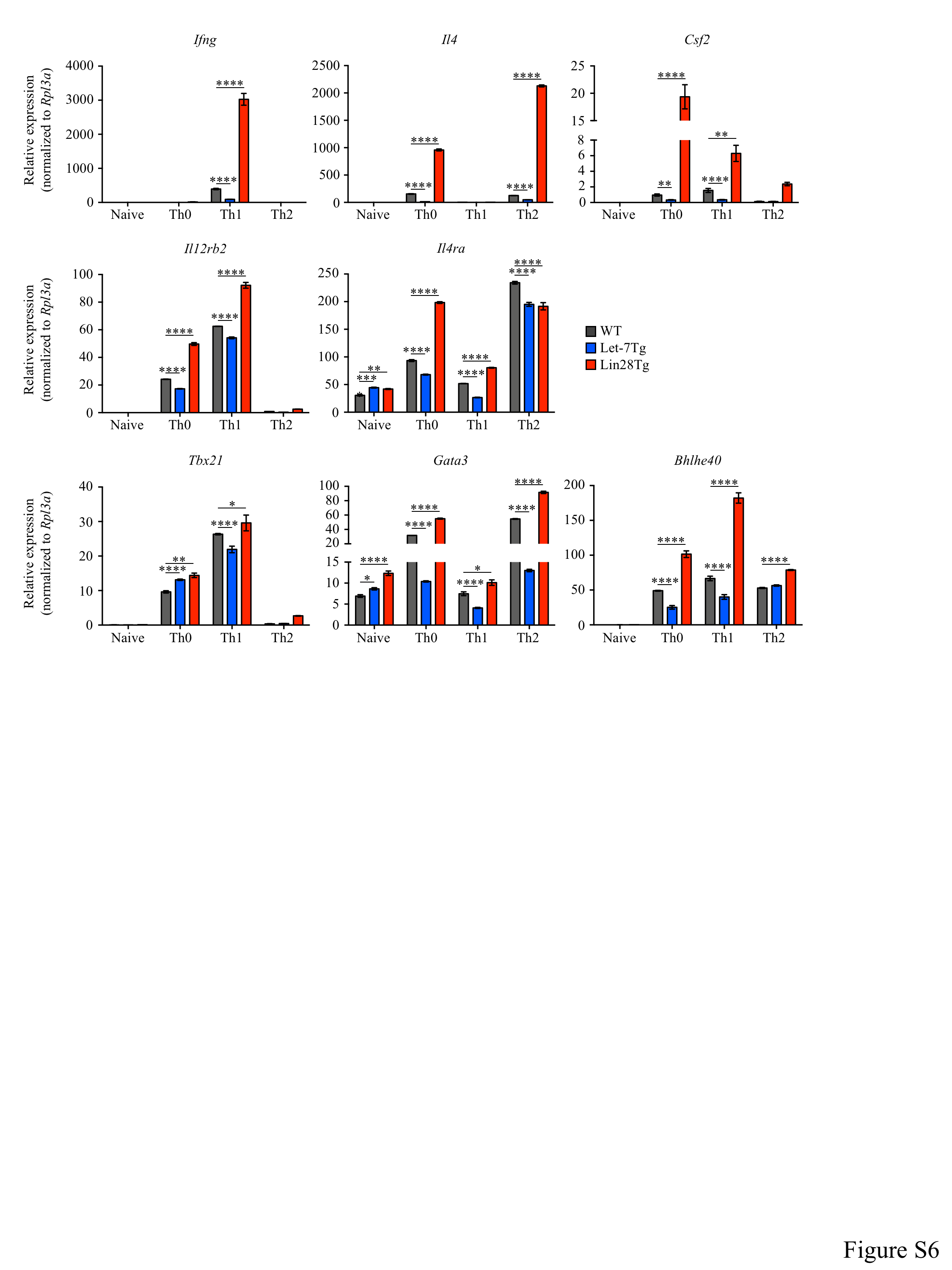

Supplement: Figure S6 — let-7 miRNAs negatively regulate the expression of genes controlling the differentiation of Th0, Th1, and Th2 cells generated in vitro. Quantitative RT-PCR analysis of the cytokines IFNγ (Ifng), GM-CSF (Csf2), and IL-4 (Il4), the cytokine receptors IL12Rβ2 (Il12rb2) and IL4Rα (Il4ra), as well as the transcription factors T-bet (Tbx21), Bhlhe40 (Bhlhe40), and GATA3 (Gata3) in naïve CD4+ T cells and in vitro-generated Th0, Th1, and Th2 cells from WT, Let-7Tg, and Lin28Tg mice, presented relative to results obtained for the ribosomal protein Rpl13a (control). *p < 0.05, **p < 0.01; ***p < 0.001, ****p < 0.0001 compared with WT using two-tailed Student's t-test. Data are from one experiment representative of two independent experiments (mean ± S.E.M. of technical triplicates of each population from all mice). [file Image_6.TIFF]

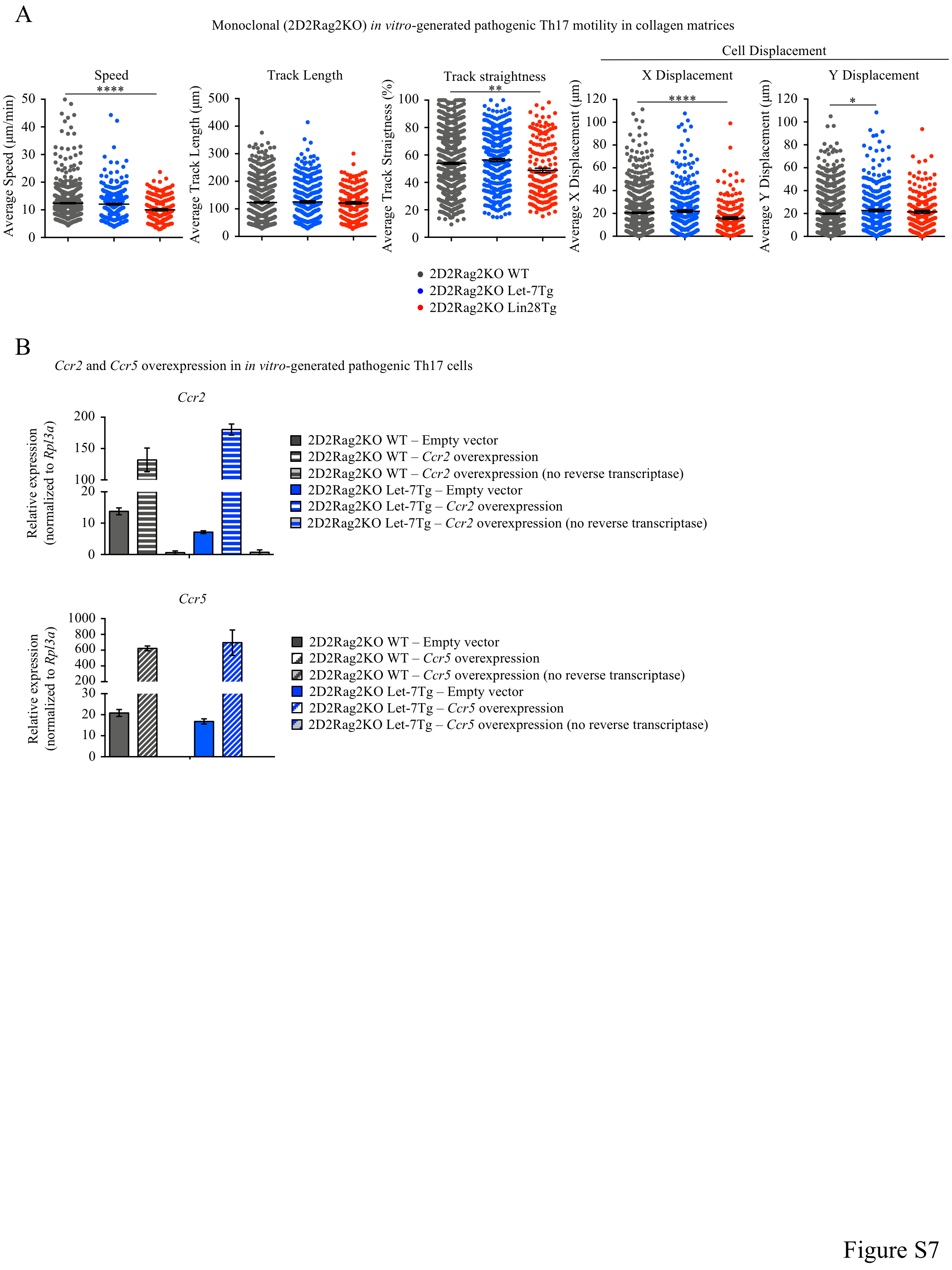

Supplement: Figure S7 — let-7 miRNAs do not control the intrinsic motility of in vitro-generated pathogenic Th17 cells. (A) Speed, track length, track straightness, and displacement of migrating CFSE-labeled in vitro-generated pathogenic Th17 cells from 2D2Rag2KO WT, 2D2Rag2KO Let-7Tg, and 2D2Rag2KO Lin28Tg mice embedded in collagen matrices. (B) Quantitative RT-PCR analysis of the chemokine receptors CCR2 (Ccr2) and CCR5 (Ccr5) in in vitro-generated pathogenic Th17 cells from 2D2Rag2KO WT and 2D2Rag2KO Let-7Tg mice, transduced with empty vector (solid bars), Ccr2-overexpression vector (horizontally-striped bars), and Ccr5-overexpression vector (diagonally-striped bars), presented relative to results obtained for the ribosomal protein Rpl13a (control). *p < 0.05, **p < 0.01, ****p < 0.0001, compared with WT using two-tailed Student's t-test. Data are from one experiment representative of two experiments (B, mean ± S.E.M. of technical triplicates of each population from all mice), or from one experiment (A, mean ± S.E.M. of at least 175 analyzed cells of each population from all mice). [file Image_7.TIFF]
